# Impact of mHealth on enhancing pre-exposure prophylaxis adherence and strengthening the HIV prevention cascade among key populations: a systematic review and meta-analysis

**DOI:** 10.3389/fpubh.2025.1600773

**Published:** 2025-06-26

**Authors:** Fan Li, Chaoying Xie, Fang Xiang

**Affiliations:** ^1^School of Nursing, University of South China, Hengyang, China; ^2^The First Hospital of Changsha, Changsha, China

**Keywords:** HIV, pre-exposure prophylaxis, mHealth, adherence, HIV prevention cascades

## Abstract

**Background:**

Good adherence to pre-exposure prophylaxis (PrEP) is critical for effective HIV prevention. Despite the growing awareness of PrEP, many individuals remain at a preliminary understanding stage and struggle to achieve sustained adherence. mHealth (mobile Health) technology is emerging as one of the promising tools in the HIV prevention cascade. While research on mHealth applications for HIV prevention is rapidly advancing, their effectiveness in promoting robust PrEP adherence and optimizing cascade outcomes remains inconclusive, with fragmented evidence limiting scalable implementation.

**Objective:**

To evaluate the efficacy of mHealth tools in optimizing the HIV prevention cascade (from risk identification to PrEP adherence) among key populations (including men who have sex with men, bisexual individuals, sex workers, transgender populations and some other groups who at elevated risk of HIV acquisition).

**Methods:**

We searched in PubMed, Cochrane Library, Web of Science, Embase, Ovid and CINAHL (EBSCO) from the inception to February 3, 2025. Our inclusion criteria focused on randomized controlled trials (RCTs). Literature screening and data extraction were performed independently by two authors. Methodological quality was assessed using Cochrane’s Risk of Bias in Randomized Controlled Trials tool. The primary outcome was adherence to PrEP and secondary outcomes included PrEP use, HIV testing and number of condomless sex events. Analyses were performed using standardized mean difference (SMD) and 95% confidence interval (CI) for continuous variables and using odds ratios (OR) and 95% CI for categorical variables. Data analysis and forest plotting were carried out using R Statistical Software version 4.4.0.

**Results:**

16 RCT studies met the inclusion criteria. The results of the meta-analysis showed that mHealth interventions significantly promoted PrEP adherence (OR = 1.60, 95% CI [1.09, 2.35], ρ = 0.016) and HIV testing (OR = 1.63, 95% CI [1.39, 1.90], ρ < 0.01). It had also shown some effectiveness in promoting the use of PrEP. However, there were no significant effects on reducing the number of condomless sex events during the entire follow-up period.

**Conclusion:**

mHealth effectively enhances specific stages of the prevention cascade. However, further optimization of technology design and intervention is needed to address complex difficulties.

**Systematic review registration:**

https://www.crd.york.ac.uk/PROSPERO/display_record.php?RecordID=533772, identifier PROSPERO CRD42024533772.

## 1 Introduction

The spread of HIV is still a serious global public health crisis, with persistent epidemiological disparities concentrated among key populations. HIV incidence among key populations, including men who have sex with men, sex workers, injection drug users, transgender people and some other groups, remains at high levels compared with the general population (1). Pre-exposure prophylaxis (PrEP) is an effective HIV prevention strategy that refers to the use of antiretroviral drugs by HIV negative individuals to prevent HIV infection. Surveillance data showing low coverage of PrEP prevention services for key populations globally in 2023 and UNAIDS is calling for greater investment in HIV prevention and social promotion programs, expanding the scale of PrEP as an effective HIV prevention measure (2). Tenofovir Disoproxil Fumarate/Emtricitabine (TDF/FTC) are currently some of the most commonly used PrEP drugs. The iPrEx trial enrolled 2,499 participants to evaluate the preventive efficacy of PrEP on HIV. Results demonstrated that daily oral PrEP (FTC-TDF) provided a 44% additional reduction in HIV acquisition risk among men who have sex with men and transgender women compared to the placebo group. Notably, participants with detectable study drug levels (indicating high adherence) exhibited a 92% lower risk of infection (95% CI, 40 to 99; *p* < 0.001). Currently, TDF and FTC have been approved in multiple countries (1, 2). Effective HIV prevention strategies are essential for controlling the spread of the virus.

There is now an increasing acceptance of the HIV prevention cascade as a framework for developing and implementing feasible prevention strategies (3). The HIV prevention cascade can improve monitoring, planning, and strengthen HIV prevention plans by identifying obstacles and inefficiencies in the HIV prevention process, integrating multiple perspectives (4). The cascade consists of three key stages: motivation, accessibility and effective use (5). Schaefer believes that individuals lacking risk perception may not have the motivation to enter the prevention cascade. Within this context, HIV testing is a critical entry point in the HIV prevention cascade. For example, HIV self-testing (HIVST) has been shown to improve early diagnosis, particularly among hard-to-reach populations. Studies have shown that the distribution of HIVST kits among men who have sex with men and transgender individuals can increase the number of tests by 1.7 and 0.82, respectively (6, 7). By enabling individuals to learn about their status in a private setting, test uptake is increased and adoption of prevention strategies is facilitated. After possessing motivation, interventions need to be provided to increase accessibility by offering products, information, or programs to the population (8). Finally, the final step of the cascade is defined as the use required to achieve protection against HIV rather than avoidance of infection, such as PrEP and condom use, and can be any prevention method or combination of methods (5, 8). This article will focus on exploring the above content.

An HIV prevention cascade approach indicates that the coverage of prevention services can be improved by targeting three key components: demand side interventions, supply side interventions and adherence interventions (4). For example, compliance interventions based on peer support and counseling support can effectively improve the coverage of prevention services. A meta-analysis included 55 studies to explore the impact of providing positive counseling support on behavioral change in men who have sex with men, of which 33 studies used mHealth technology. The results showed that after receiving counseling support, 100% of participants exhibited a reduction in sexual risk behavior and approximately 27% started PrEP, indicating a positive impact of consistent support on the use of prevention strategies (9). However, substantial gaps remain in the effective use phase of the prevention cascade: despite the increase in PrEP use, 41% of the participants discontinued PrEP within 6 months and those who continued to use PrEP were unable to achieve good adherence, which will result in limited effectiveness of PrEP for prevention (10); and testing and counseling services continue to be insufficient-covered in resource-constrained areas, where individuals do not receive expected testing services, which has a negative effect on HIV prevention (11).

The integration of mHealth technology into HIV care has come a long way with advances in technology. mHealth encompasses delivering health information and services via the Internet, wireless technology and various digital technologies, such as phone calls, text messages, emails, short videos, social media and wireless device (12, 13). Research indicates that mHealth has shown promising potential in improving adherence to antiretroviral therapy (ART) among person living with HIV (14, 15), reducing the risk of HIV transmission, as well as enhancing the quality of life of individuals. The integration of mHealth technology into interventions can increase users’ awareness of PrEP (16), assist in overcoming challenges to accessing and adhering to PrEP and enhance the ability to manage sexual health independently (17). Several scoping reviews have demonstrated the potential effectiveness and feasibility of mHealth for PrEP adherence (18, 19). However, to the best of our knowledge, there are few studies evaluating the impact of mHealth-based interventions on the entire HIV prevention cascade. And based on the potential limitations of observational studies, we only included the results of randomized controlled trials. This study aimed to summarize the various forms and components of mHealth interventions and evaluate the impact on the effective use of HIV prevention methods among people at risk of acquiring HIV.

## 2 Methods

### 2.1 Search strategy

This study adhered to the Preferred Reporting Items for Systematic Reviews and Meta-Analyses (PRISMA) 2020 guidelines (20). Two authors conducted a comprehensive search of the PubMed, Cochrane Library, Web of Science, Embase, Ovid, CINAHL (EBSCO) and China National Knowledge Infrastructure (CNKI) from inception to February 3, 2025 and the type of literature included was randomized controlled trials (RCTs) with no language restrictions. The main content of the search included: AIDS, mHealth interventions, pre-exposure prophylaxis, medication adherence, awareness, condom and HIV testing. We also manually retrieved and supplemented the list of relevant references without including literature such as conference abstracts and dissertations. Details of the electronic search strategy were shown in Supplementary material 1. The study was pre-registered in PROSPERO (CRD42024533772). All data were obtained from published articles and no ethical approval was required.

### 2.2 Selection criteria

The following quality criteria were developed in accordance with the PICOS principles.

#### 2.2.1 Participants

There were no regional restrictions on the inclusion of the population. The eligible participants were required to be HIV-seronegative individuals at elevated risk of HIV acquisition, including men who have sex with men, bisexual individuals, sex workers, transgender populations and some other groups. People living with HIV were excluded.

#### 2.2.2 Intervention

Interventions were defined as the provision of HIV prevention services through wireless technology or mobile devices, such as text messaging, phone calls, emails, videos, games, websites, social media and wireless equipment.

#### 2.2.3 Comparison

Most of the control group received usual care. Some of the controls took the original study-defined waitlist or attention-matched controls.

#### 2.2.4 Outcome

The primary outcome was good adherence to PrEP, which was defined as dried blood spot (DBS) tenofovir diphosphate (TFV-DP) concentrations of > 700 fmol/punch or ≥ 4 days of PrEP use per week. Secondary outcomes included PrEP use, HIV testing behaviors and number of condomless sex events. PrEP use was defined as the proportion of people who used PrEP at least once based on any form of report during follow-up. HIV testing was defined as the proportion of participants who participated in HIV testing (at least once) during the follow-up period. All forms of PrEP medication included in this study were oral PrEP. Non-oral forms such as the Vaginal Ring for HIV Prophylaxis, Subdermal Implants and Long-Acting Injectable Pre-Exposure Prophylaxis (LAI-PrEP) were excluded. All outcome indicators were required to include at least short-term (12 weeks) or long-term (24 weeks) evaluations.

#### 2.2.5 Study

All included studies were randomized controlled trials (RCTs).

### 2.3 Study selection and data extraction

Literature screening was performed using the literature management program EndNote X9 3.3. We excluded the following studies: duplicate publications, conference abstracts and articles for which full text or complete data were not available. Initial screening involved independent assessment of titles and abstracts by two authors based on eligibility criteria, with any discrepancies resolved through consultation with a third researcher. Data extraction utilized a predefined form in Microsoft Excel, capturing details including first author, publication year, country, patient demographics, sample size, intervention program overview, control group details and outcome indicators. If data were missing, we would attempt to contact the authors for details.

### 2.4 Quality assessment

Literature quality assessment was conducted independently by two researchers according to the Cochrane Handbook for Systematic Reviews of Interventions (21). Evaluations were made on seven areas: random sequence generation, allocation concealment, blinding of participants and personnel, blinding of outcome assessment, incomplete outcome data, selective reporting and other potential biases. Each area was rated as having a low, unclear or high risk of bias. Any disagreements were resolved through consultation with a third review panel member. The overall quality of evidence was appraised using the Grading of Recommendations Assessment, Development and Evaluation (GRADE) system (22). The initial evidence quality of RCT was high-quality evidence. Possible factors that reduced the quality of evidence included: risk of bias, inconsistency, indirectness, imprecision and publication bias. After evaluation, the final level of evidence quality was divided into high, medium, low and very low.

### 2.5 Statistical analysis

The study population and intervention characteristics of each study were summarized in the form of textual descriptions, with summary results presented in tabular form. Meta-analyses, heterogeneity tests and sensitivity analyses were conducted using the “metafor” package in R Statistical Software version 4.4.0 (23). Continuous variables were combined using standardized mean difference (SMD) with a 95% confidence interval (CI). Categorical variables were analyzed by first combining outcome data and calculating the odds ratio (OR) with a 95% CI. Statistical heterogeneity between studies was assessed using the I^2^ statistic and Cochran’s Q Test; I^2^ ≤ 50% and *p* > 0.1 indicated no significant heterogeneity, allowing the use of a fixed-effects model. Significant heterogeneity (I^2^ > 50% or *p* < 0.1) warranted the use of a random-effects model. Subgroup analyses and meta-regression were not performed due to the limited number of included studies. All *p*-values were two-sided, with ρ < 0.05 indicating statistical significance. In forest plots, each study was represented by a block, with its size intuitively indicating the study’s weight. This referred to the contribution of an individual study to the overall results, with studies having larger sample sizes assigned higher weights. Larger weights corresponded to greater impacts on the pooled effect.

## 3 Results

### 3.1 Search results

**Figure 1:**
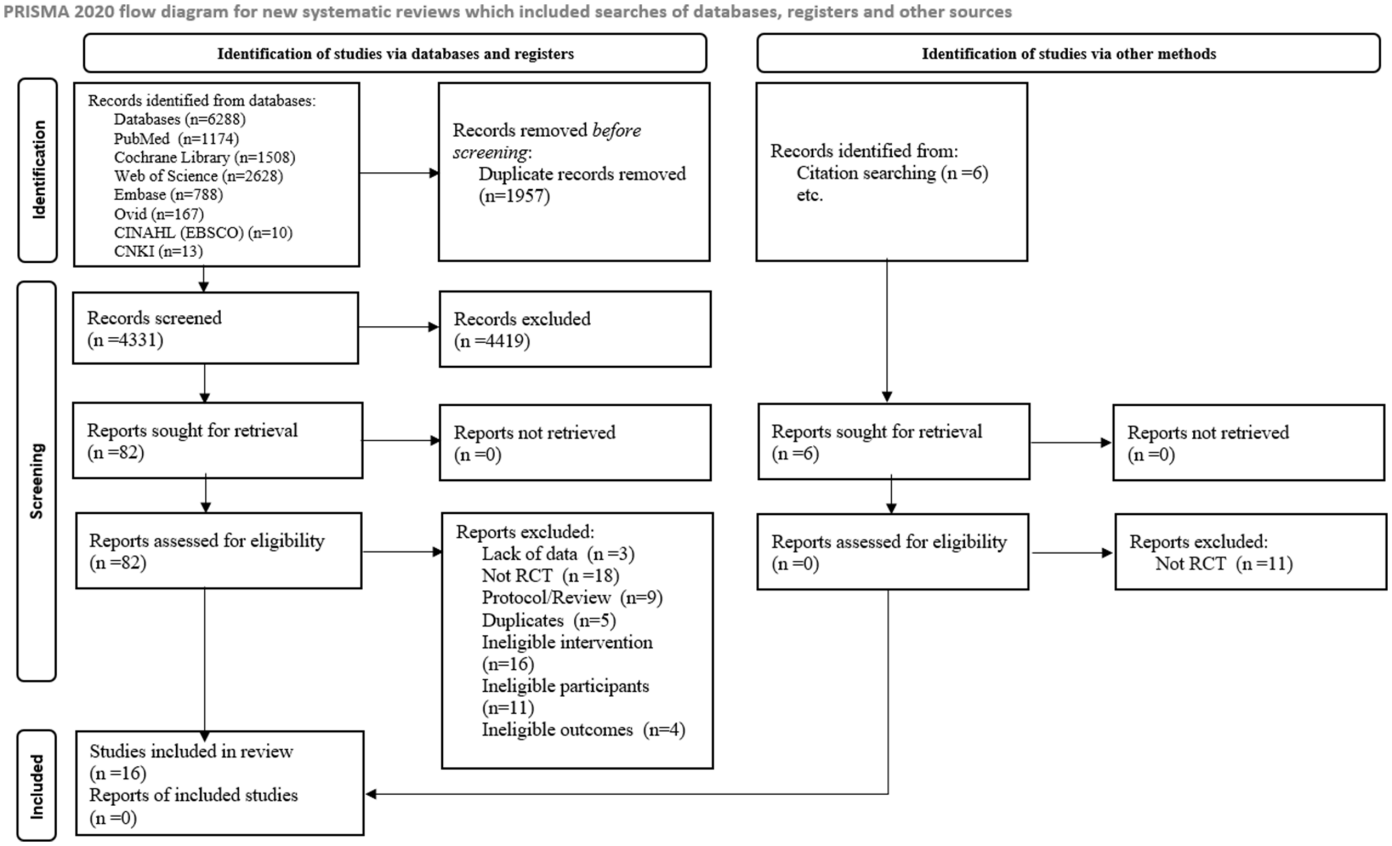
PRISMA 2020 flow diagram.

An initial search from 7 databases yielded 6,288 studies, 4,331 studies were obtained after excluding 1,957 duplicates and 82 studies were obtained after reading titles and abstracts. 16 studies were finally included after reading the full text. Additionally, 6 studies were retrieved through citation searching, with no study meeting the inclusion criteria. A total of 16 studies were finally included and the detailed flowchart of the literature screening process is provided in Figure 1.

### 3.2 Study characteristics

A total of 16 studies involving 7,097 participants, all RCTs reported in English with publication dates between 2018 and 2025. Study areas including the United States, Thailand, Kenya, China, South Africa, Zimbabwe and Mozambique were included in this meta-analysis. Detailed characteristics were shown in Table 1.

**Table 1:** The characteristics of included studies.

Author, publication year	Country	Type of patients	Number of subject (intervention/control)	Mean age of subject	Intervention group					Control	Outcome measures
					Name	Intervention introduction	Theory	Format	Duration		
Moore 2018 (24)	America	(1) (2)	398 (200/198)	35.2	iTAB	Receive a personalized, two-way, fully automated text message based on SoC^d^.	Behavioral theory	Text message	48 weeks	SoC	①
Liu 2019 (25)	America	(1)	121 (81/40)	24.2	PrEPmate	Includes weekly “check-in” messages and customized text messages to send medication reminders.	IMB	Text message	36 weeks	SoC	①
Bauermeister 2019 (33)	America	(3)	180 (120/60)	21.5	myDEX	The program comprises six sessions, encompassing diverse cognitive and emotional content domains, and provides access to relevant activities and videos.	cognitive-affective dual-process models	Mobile APP	12 weeks	Attention-matched control	②③
Njuguna 2019 (36)	Kenya	(4)	600 (300/300)	21	SMS	Participants will receive information about HIV and reproductive health topics every week, and can also request other messages on the same topic, allowing them to browse menus on other topics.	-	Text message	24 weeks	SoC	③
Zhu 2019 (29)	China	(1)	100 (50/50)	-^c^	WeTest	It provides users with information about the use and explanation of the HIVST test kit, as well as other information about HIV transmission, other STI risks, reducing behavioral risks, and the importance of regular HIV testing.	IMB	Mobile APP	24 weeks	SoC	③
Kaymarlin 2019 (37)	South Africa, Zimbabwe and Mozambique	(5)	1,783 (960/823)	-^c^	SMS	Participants received brief messages. It focuses on promoting the continuous use of condoms, reducing the number of sexual partners and advocating regular HIV testing.	-	Text message	24 weeks	SoC	③
Songtaweesin 2020 (26)	Thailand	(1) (2)	200 (100/100)	18	APP+YFS	Users can enter relevant data each week to calculate HIV risk values and receive rewards based on behaviors such as data entry and responding to follow-up calls.	IMB	Mobile APP	24 weeks	YFS	①
Haberer 2021 (34)	Kenya	(4)	348 (173/175)	21	MPYA	Text messages will be sent every day in the first month. After 1 month, participants can personalize the text message content and sending frequency.	-	Text message	24 weeks	SoC	②
Whiteley 2021 (27)	America	(1)	69^a^	25.1	ViralComba	Learn about HIV and medications through games and scrolling messages to get information from health care providers.	IMB	Game	24 weeks	Attention-matched control	①
Schnall 2022 (32)	America	(1)	763 (382/381)	16.2	MyPEEPS Mobile	Contains 21 online psychoeducation and skill-building modules to train participants in condom use, emotion regulation and communication skills, and prompts for improvement goals (building HIV knowledge, self-awareness and self-efficacy).	Social learning theory	Mobile APP	12 weeks	Waitlist	②③④
Sullivan 2022 (31)	America	(3)	837^b^	-^c^	M-cubed	It provides participants with customized preventive services such as written and video messages, with regular push functionality.	Social cognitive theory	Mobile APP	12 weeks	Waitlist	①②③
Lin 2023 (38)	China	(1)	935 (404/531)	26	Multilevel Intervention	Distribute digital materials and provide HIV self-testing service information through WeChat, and establish online groups to discuss HIV prevention.	-	Mobile APP	-^c^	SoC	③
Erenrich 2024 (39)	America	(1) (2)	229 (116/113)	23.7	PrEPTECH	Provide online PrEP education, and obtain customized PrEP prescription guidance and regular testing services by filling out the medical questionnaire on the platform.	-	Online website	24 weeks	Soc	②④
Wray 2022 (35)	America	(1)	73 (37/36)	35.4	GAME PLAN	A web- and SMS-based intervention with customizable plans to increase PrEP use, reduce condomless sex events and drinking, and set SMS reminders.	-	Online Website	24 weeks	Attention-matched control	④
Horvath 2024 (28)	America	(1)	80 (40/40)	25.1	PrEP iT	A smartphone-based program equipped with self-monitoring, dynamic information reporting, and expert consultation.	IMB	Mobile APP	24 weeks	Soc	①②
Biello 2025 (30)	America	(3)	381 (251/130)	22.4	Mychoice/LYNX	“Mychoice: Recommend health information such as infographics, videos, educational resources and provide personalized HIV testing plans and PrEP./ LYNX: An app that can provide sexual diary, HIV or STI testing reminders; Order household HIV and STI testing kits, a PrEP information page, including a roadmap on how to obtain PrEP; Provide chat functions, etc.	Social cognitive theory/IMB	Mobile APP	24 weeks	Soc	②③

Type of patients: (1) Men who have sex with men. (2) Transgender women. (3) Gay, bisexual and other men who have sex with men. (4) Young women. (5) Other groups at high risk of HIV acquisition. Outcome Measures: ① Good adherence to PrEP. ② PrEP use. ③ HIV testing. ④ Condomless sex events. ^a^The article did not specify the specific number of intervention and control groups and the randomization ratio. ^b^The article did not specify the specific number of intervention and control groups but only reported the randomization ratio. ^c^Not reported. ^d^Standard of care.

Among the 16 studies, mHealth interventions were in the form of text messages (5 studies), mobile applications (8 studies), games (1 study) and online websites (2 studies). The research was based on theories such as Behavioral theory (24), Information-Motivation-Behavioral Skills Model (IMB) (25–30), Social cognitive theory (30, 31), Social learning theory (32) and cognitive-affective dual-process models (33). These interventions were classified into four categories based on the nature of the intervention: sending PrEP medication reminders (24, 25, 34, 35), setting up PrEP incentives (26), providing relevant HIV and PrEP knowledge (27, 29–33, 36–38) and offering personalized PrEP guidance services (28, 36, 39).

Participants were all HIV-negative individuals including men who have sex with men (24–33, 35, 38, 39), transgender people (24, 26, 39), bisexuals (30, 31, 33), young women with limited access to sexual health resources (34, 36) and other groups (37). In an in-depth analysis of Sullivan’s (31) study, we observed differences in the study population in the data. In order to better parse the impact of the study on the conclusions, we decided to split the data from this literature into two parts for meta-analysis according to the study population: groups with low adherence to condom use or PrEP use (Represented by 2022a) and groups of adherent PrEP users (Represented by 2022b). In Biello’s (30) study, a three-arm control was used and two intervention measures were set up to compare with the control group. We will set the “Mychoice group” to “2025a” and the “LYNX group” to “2025b.”

### 3.3 Quality assessments of included studies

**Figure 2:**
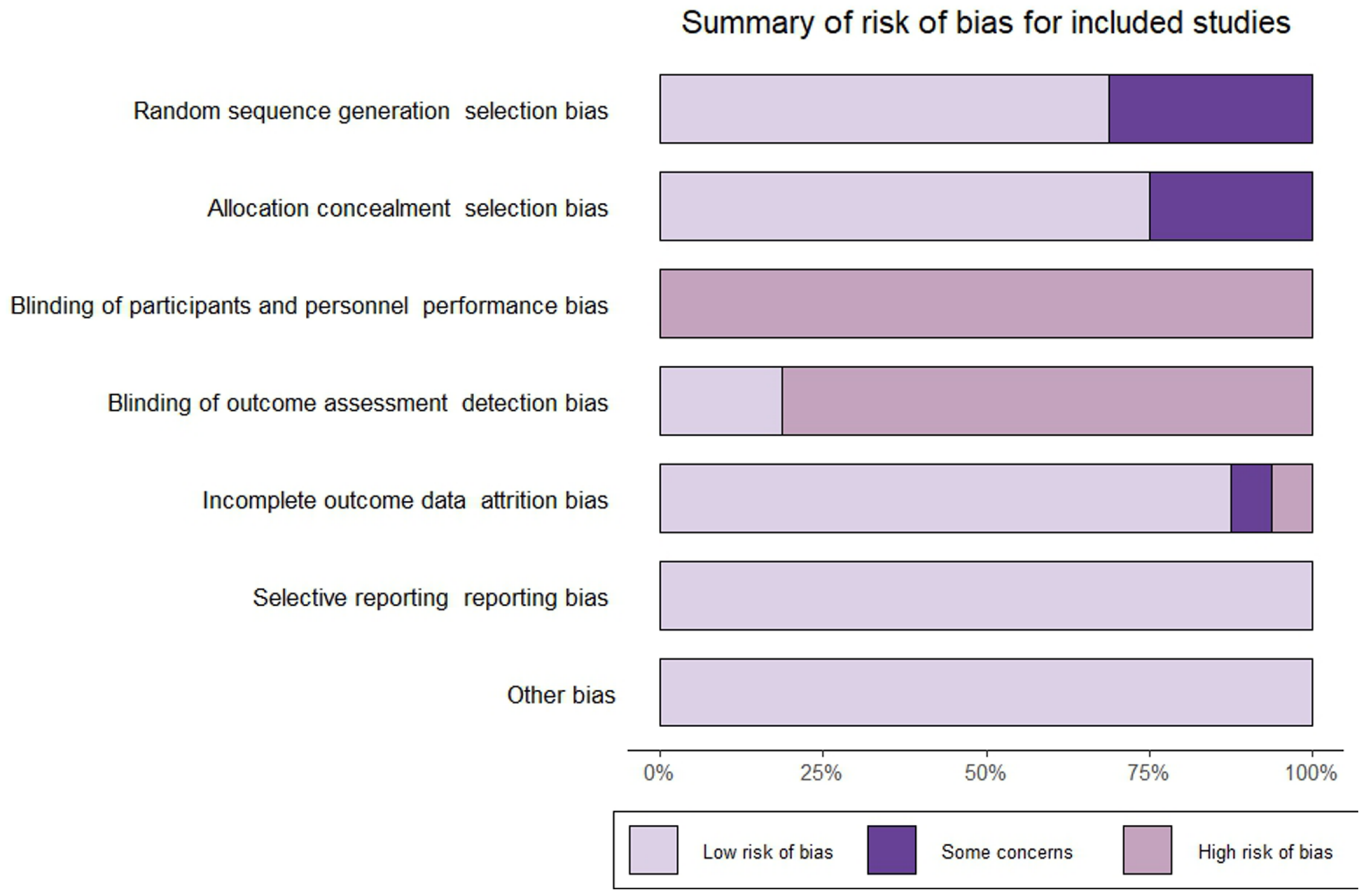
Risk assessment of bias.

Five studies (24–26, 28, 39) did not report randomized sequence generation and four studies (26, 28, 36, 39) did not report allocation concealment, resulting in these studies being judged as having “some concerns” about selection bias. Based on the nature of the intervention, all studies were judged to be at “high risk” of not being able to blind participants and performers. Three studies (24, 25, 27) included blinding of outcome measures and were rated as having a “low risk” of bias. Due to an imbalance in the number and reasons for missing data between groups, a study (31) was rated as “high risk” in the field of “incomplete outcome data.” And one study (27) was rated as “some concerns” in the “incomplete outcome data” domain due to the lack of reporting on the reasons for participant dropout. All studies were rated as “low risk” in “selective reporting” and “other biases.” We used the “Rob” package to produce images for risk of bias assessment and methodological quality results were shown in Figure 2.

The quality of evidence for all outcome indicators in this study was assessed using the GRADE approach. The evidence quality was rated as moderate for good PrEP adherence, very low for PrEP use, low for HIV testing and moderate for condomless sex events. Detailed evaluation results were presented in Supplementary material 2, with footnotes identifying detailed reasons for downgrading. Downgrades occurred primarily due to issues with allocation concealment and unclear random sequence generation, with some studies further downgraded for high statistical heterogeneity or imprecision. No studies were downgraded due to indirectness.

### 3.4 Effects of mHealth on PrEP adherence

**Figure 3:**
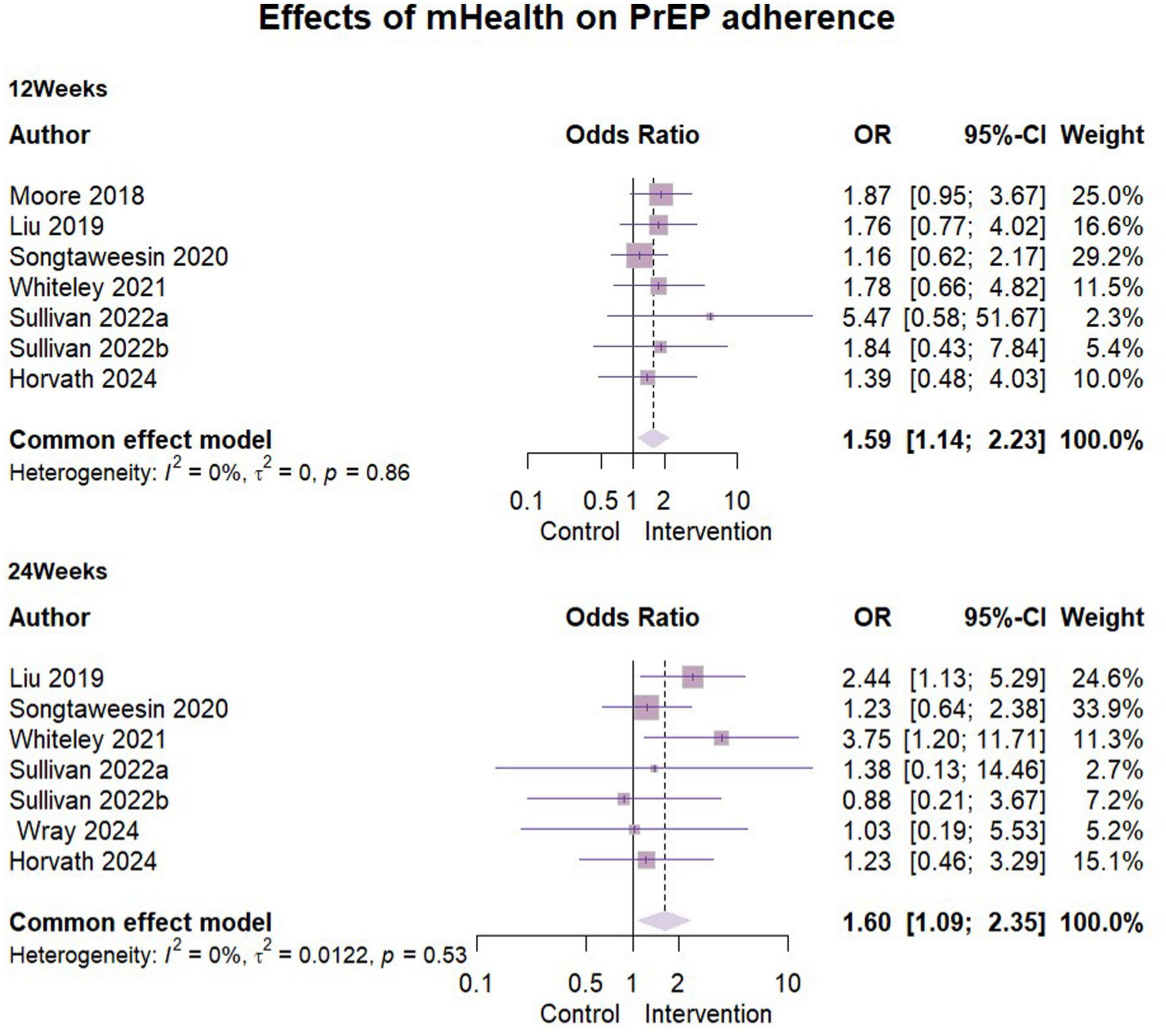
Effects of mHealth on PrEP adherence.

A total of seven studies (24–28, 31, 35) reported the results of mHealth interventions on PrEP good adherence, with *n* = 1,617 at 3 months follow-up and *n* = 1,301 at 6 months follow-up. One study (31) used self-reported results, while other studies used the dry blood spot (DBS) concentration of tenofovir diphosphate (TFV-DP) at the visit as a measure of good adherence to PrEP. The results showed statistically low heterogeneity in all of them and the ORs were combined using a fixed-effects model. The detailed data were presented in the forest plots (Figure 3). We found that the mHealth intervention significantly contributed to the development of good PrEP adherence among key populations, with statistically significant results at 3 months’ follow-up (OR = 1.59, 95% CI [1.14, 2.23], ρ = 0.007) and at 6 months’ follow-up (OR = 1.6017, 95% CI [1.09, 2.35], ρ = 0.016).

### 3.5 Effects of mHealth on HIV testing

**Figure 4:**
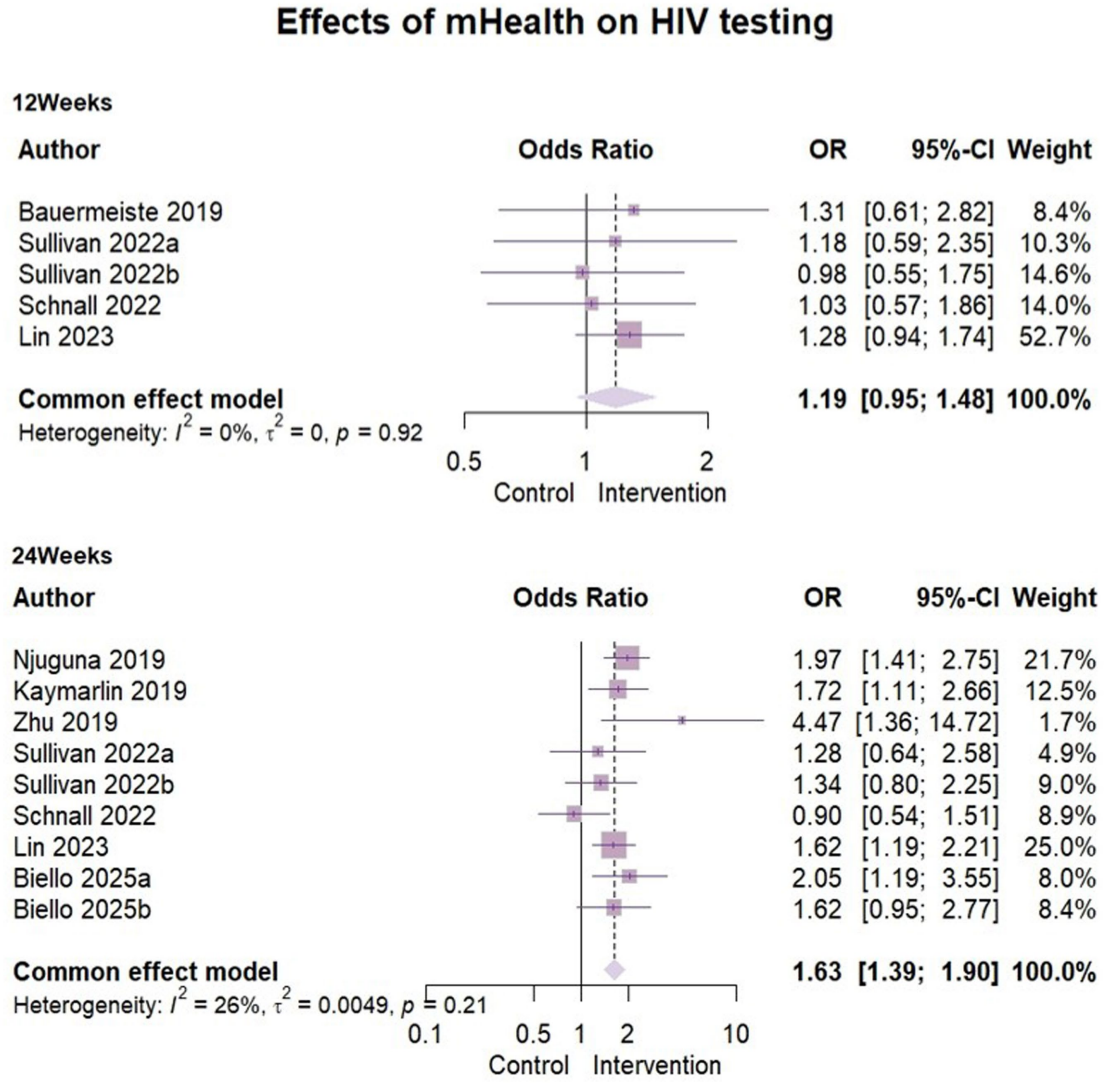
Effects of mHealth on HIV testing.

Eight papers (29, 30, 33, 36–38) reported outcome indicators of HIV testing behavior. The analysis included 2,715 participants at the 3-month assessment and 5,399 at the 6-month assessment. Heterogeneity was low at both stages (3-month: I^2^ = 0%, ρ = 0.92; 6-month: I^2^ = 26%, ρ = 0.21), supporting the use of fixed-effects models. The results of mHealth on increasing HIV testing were as follows: compared to 3 months’ follow-up (OR = 1.19, 95% CI [0.95, 1.48], ρ = 0.13), the effect size showed a significant effect at 6 months (OR = 1.63, 95% CI [1.39, 1.90], ρ < 0.01). This suggested that mHealth intervention may have some positive effects on promoting HIV testing behavior (Figure 4).

### 3.6 Effects of mHealth on PrEP use

**Figure 5:**
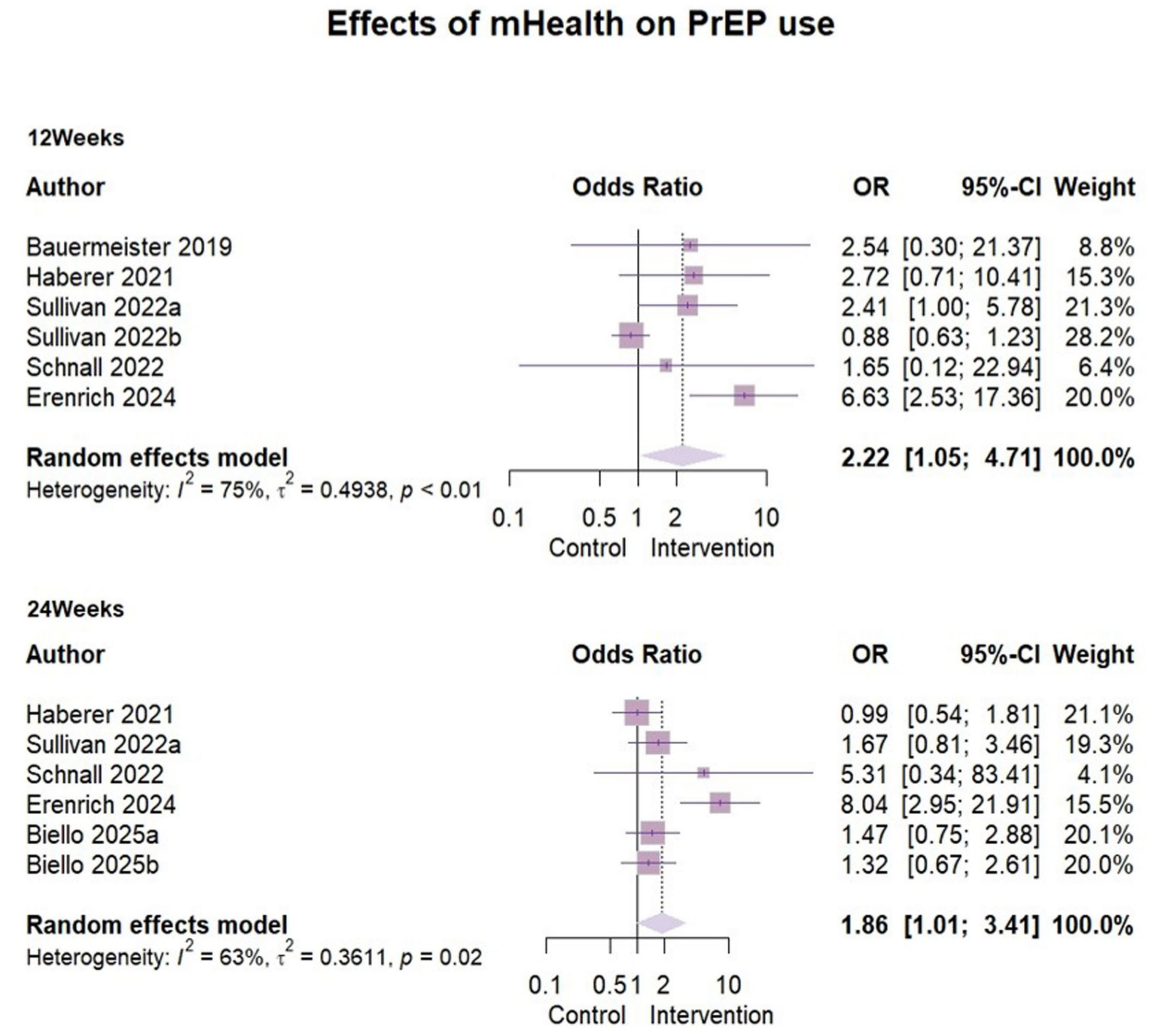
Effects of mHealth on PrEP use.

Six studies (30–34, 39) reported the effect of mHealth interventions on PrEP use (Figure 5), with *n* = 2,357 at 3 months follow-up and *n* = 2,558 at 6 months follow-up.

Due to significant heterogeneity (I^2^ = 75%, ρ < 0.01), a random-effects model was used for analysis. The effect size odds ratio (OR) at 3 months follow-up was 2.22 (95% CI [1.05, 4.71]). Because of the limited number of studies, meta-regression, subgroup analyses could not be performed to look for heterogeneity. However, we found some clinical heterogeneity. The specific reasons were outlined in the sensitivity analysis below and here we excluded Sullivan’s (31) data (Represented by 2022b) when analyzing the effect of six-month follow-up. The results indicated a high degree of heterogeneity (I^2^ = 63%, ρ = 0.02) as before. Given the limited number of included studies (*n* = 5), subgroup analyses could not be conducted due to insufficient sample size. We analyzed the study area, study design and baseline characteristics of the sample, but were still unable to identify sources of heterogeneity. A random effects model was used to report the overall effect size, OR = 1.86 (95%CI [1.01, 3.41]).

### 3.7 Effects of mHealth on condomless sex events

**Figure 6:**
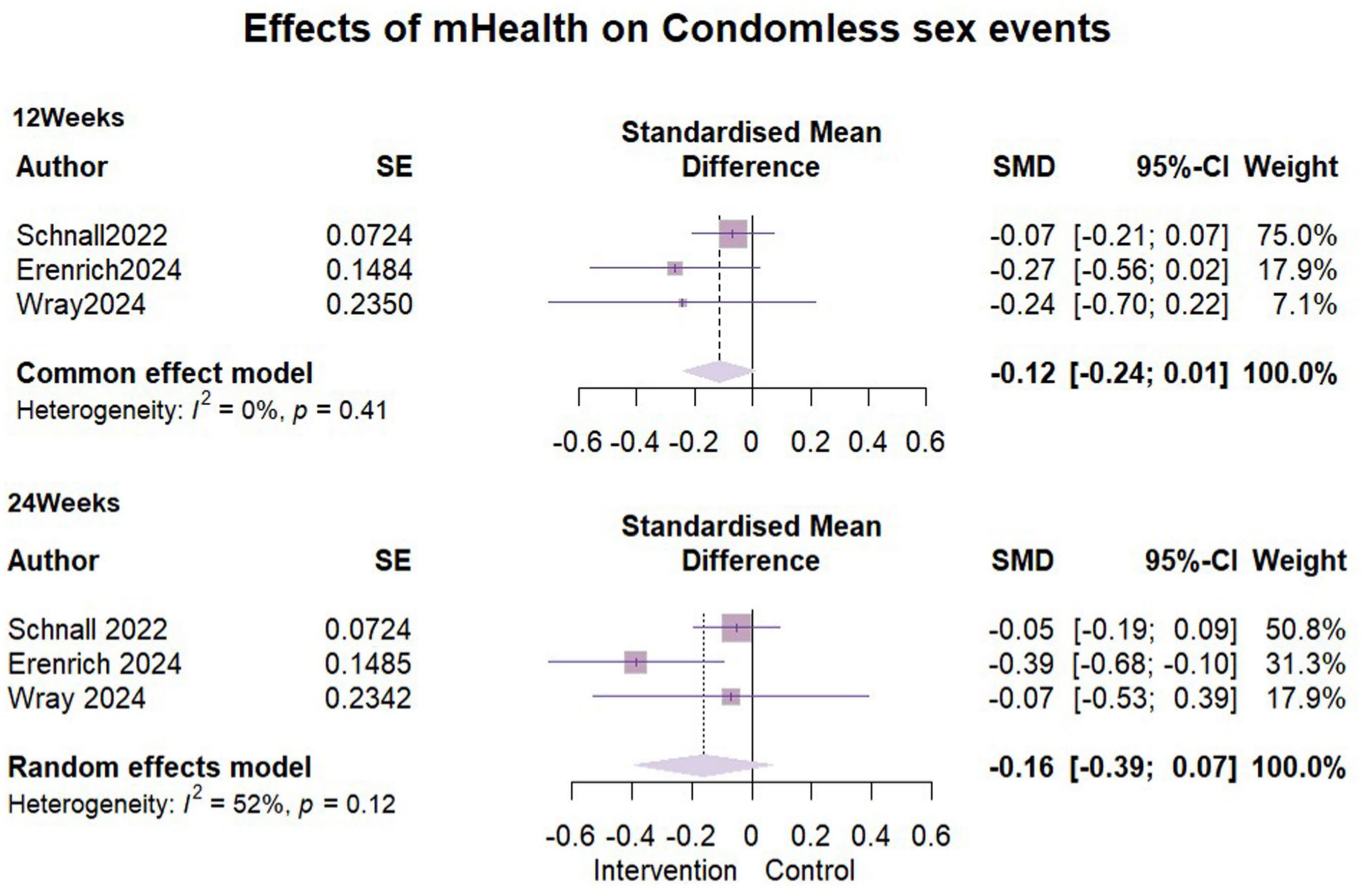
Effects of mHealth on condomless sex events.

We incorporated three studies (32, 35, 39) to compare the impact of mHealth interventions on condomless sex events with exactly 1,065 participants included in each of the two follow-ups. At the three-month follow-up, no heterogeneity was detected (I^2^ = 0%, ρ = 0.41), so a fixed-effects model was used. Pooled results showed that there was no significant difference in the number of condomless sex events between the intervention and control groups in the short term (SMD = −0.12, 95% CI [−0.24, 0.01]). At 6 months follow-up, moderate statistical heterogeneity was detected between the study groups (I^2^ = 52%, ρ = 0.12). Despite efforts, the source of heterogeneity could not be identified, and a random-effects model was applied. This showed an SMD = −0.16, (95% CI [−0.39, 0.07]), indicating no significant difference similarly between the groups at 6 months follow-up. Detailed results are presented in Figure 6.

### 3.8 Sensitivity analysis and publication bias analysis

**Figure 7:**
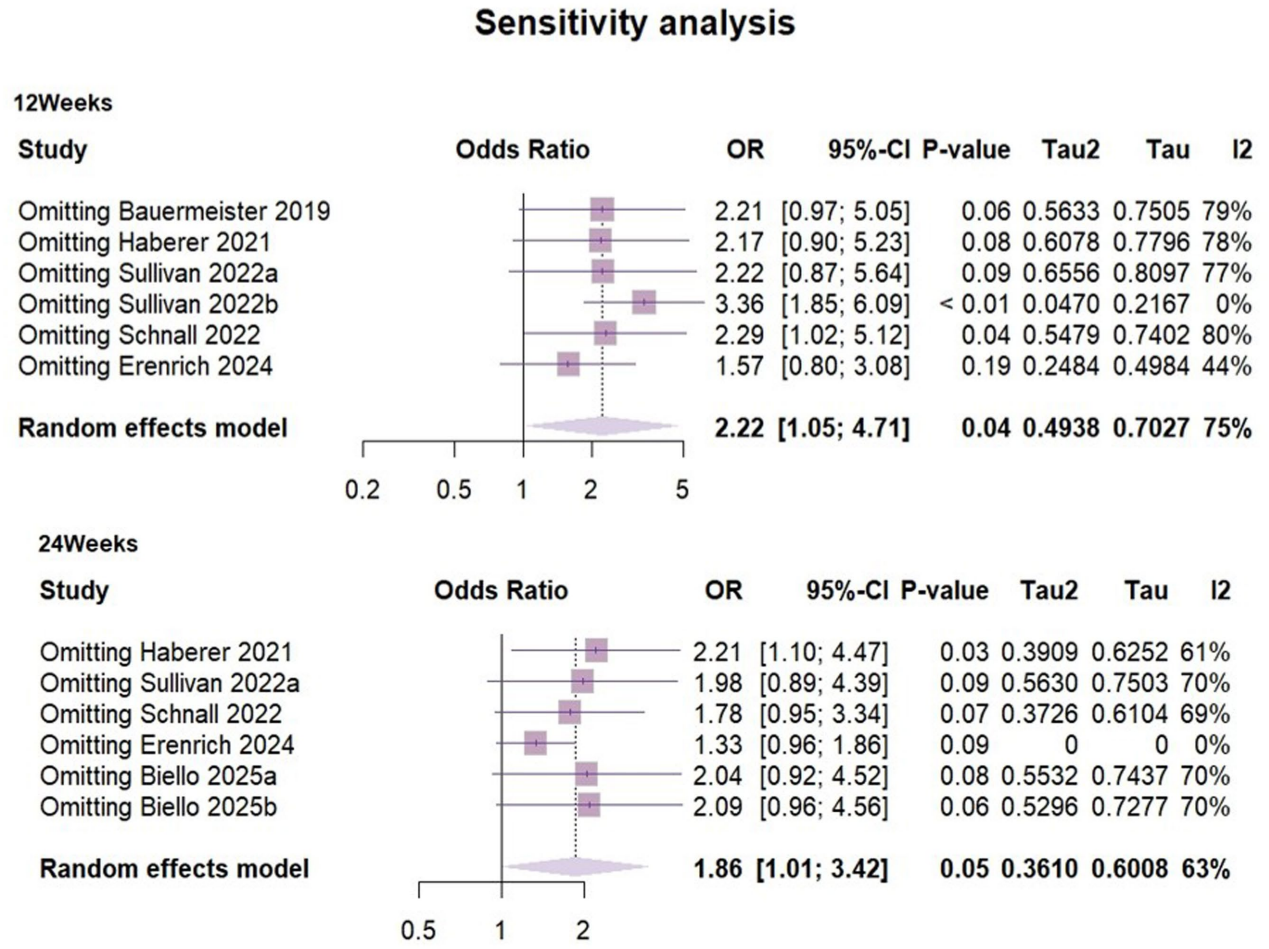
Sensitivity analysis.

Heterogeneity in both short-term outcomes and long-term outcomes of PrEP use was relatively high, so sensitivity analyses were performed (Figure 7). The results suggested that at 3 months of follow-up, groups of adherent PrEP users in Sullivan’s (31) study may have been the outlier study, and a source of heterogeneity.

To explore potential sources of heterogeneity, we compared sample characteristics, study designs and interventions. We identified that some participants in Sullivan’s (31) study (Represented by 2022b) exhibited good preventive practices, meaning they used PrEP or condoms for all sexual activities in the past 3 months. This group (gay, bisexual and other men who have sex with men) already demonstrated high levels of HIV prevention awareness and consistent health behaviors at baseline, with more regular PrEP use compared to other participants. Therefore, there were reasonable grounds to believe that there was a considerable clinical heterogeneity between this group and the other study subjects. Re-performing the meta-analysis after excluding the sample from this group, the I^2^ value decreased to 0% and ρ = 0.42. The combined OR = 3.38 (95%CI [1.95, 5.86], ρ < 0.01), This suggested that the mHealth intervention significantly increased PrEP use among people who having a risk of exposure to HIV during the three-month follow-up period.

At the six-month follow-up, the sensitivity analysis suggested that Erenrich’s study (39) could be a source of heterogeneity. Due to the limited number of existing literature, subgroup analysis was not conducted in this study. Although we compared factors such as participant type, age, intervention measures and outcome measurement methods, we still could not determine a clear explanatory factor.

After re-evaluating the result by excluding this study, the effect size shifted from 1.86 (95%CI [1.01,3.41]) to 1.33 (95CI% [0.96, 1.86]) with reduced heterogeneity (I^2^ = 0). Due to significant changes in the results of the remaining studies, our findings were unstable. It was worth noting that the exclusion of studies must be based on pre-set criteria and were only used for sensitivity analysis to evaluate the robustness of results, but should not be used as the primary basis for conclusions. Although there were potential signals of intervention effectiveness (OR = 1.86, ρ = 0.046), high heterogeneity and limited number of studies hinder clear conclusions. mHealth interventions showed a potential positive impact on improving the long-term effect of PrEP use. However there was a high degree of uncertainty. Future research should include more similar studies and larger-scale randomized controlled trials to further validate the findings.

**Figure 8:**
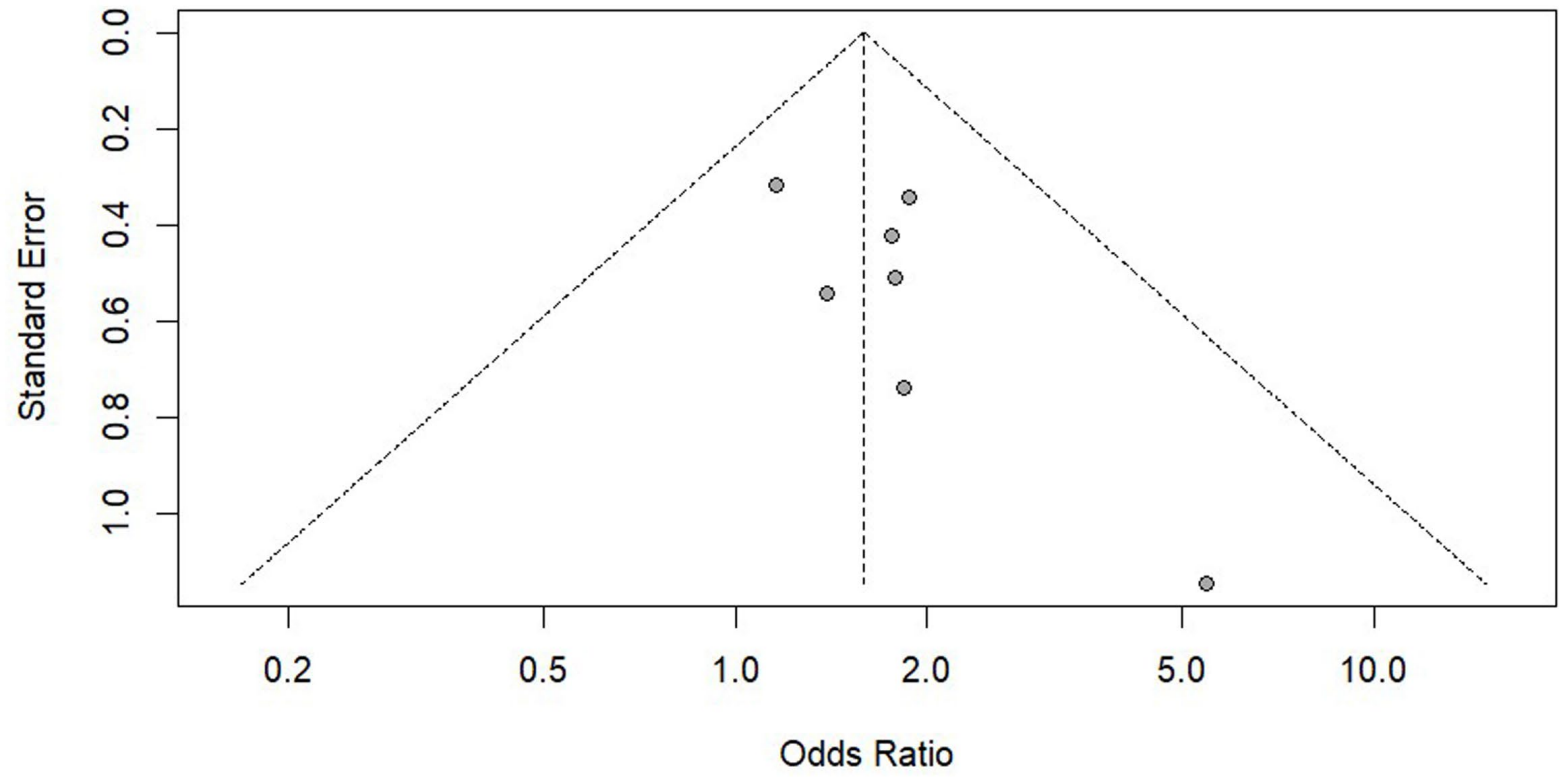
The funnel plot of PrEP adherence at three month.

**Figure 9:**
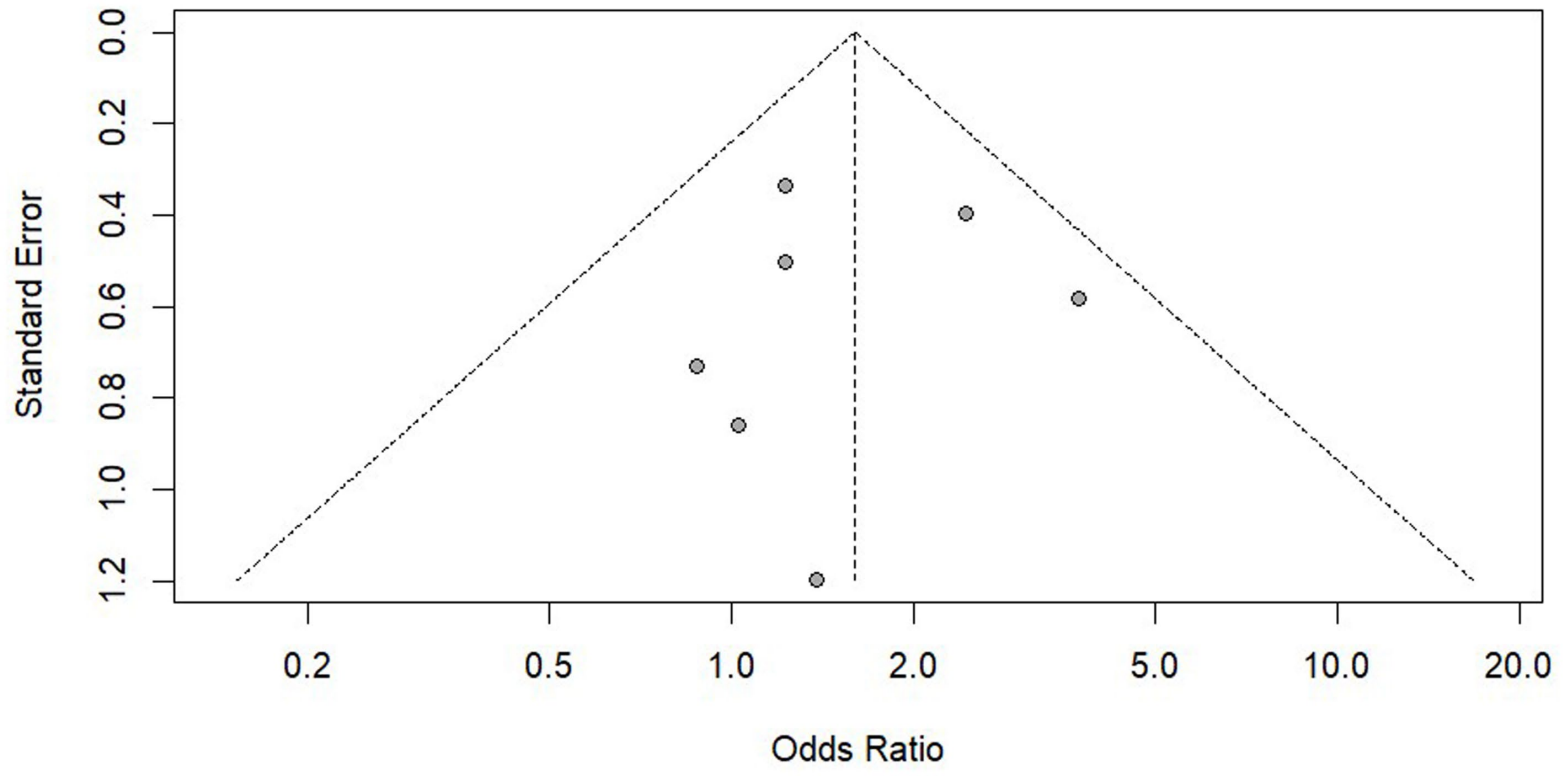
The funnel plot of PrEP adherence at six month.

We conducted a publication bias analysis on the results of PrEP adherence and reported the results using funnel plots (Figures 8, 9). According to the suggestion of Pustejovsky (40), it was beneficial to test the publication of biased and selective results. A modified Egger’s regression test (41), which substitutes standard errors with the square root of study sample sizes (robust across all proportional outcomes), was applied to PrEP adherence outcomes at different follow-up timepoints. No significant funnel plot asymmetry was detected (ρ = 0.104, ρ = 0.811).

The 3-parameter selection model (3PSM) was applied for validation, using the likelihood ratio test (LRT) to estimate the proportion of non-significant to significant results based on an alpha threshold (e.g., 0.05). A significant LRT result indicated selective reporting based on statistical significance. The analysis was performed using the “selmodel” function in the “metafor” package. Non-significant LRT results (*p* = 0.128, *p* = 0.064) were obtained, suggesting no statistically significant evidence of selective reporting.

The same methods were used to analyze the use of PrEP and HIV testing and no significant publication bias was found. However, as the statistical evidence did not reach conventional significance thresholds and the included studies were limited in number (k = 7), the statistical power might be insufficient. It was recommended to verify this through large sample studies in the future.

## 4 Discussion

This meta-analysis included 16 randomized controlled trials with a total of 7,097 participants, investigating the impact of mHealth interventions on pre-exposure prophylaxis (PrEP) adherence and HIV prevention behaviors in people at risk of acquiring HIV. We found that the mHealth intervention had a significant impact on the development of good PrEP adherence and HIV testing among key populations. While mHealth interventions showed potential to promote PrEP use, their long-term effectiveness demonstrated significant instability. Furthermore, these mHealth interventions did not significantly reduce condomless sexual events. Given the very low or low certainty of some outcome evidence, further high-quality randomized controlled trials are needed to draw more reliable conclusions. Among the included studies, only one author (31) assessed PrEP adherence through self-reporting, while nearly all studies relied on self-reported data to measure both PrEP use and condomless sex events. However, self-reported outcomes may not accurately reflect reality due to potential recall bias and intentional misreporting (42, 43). This may also be the reason for the instability of the PrEP use results we obtained. Currently, objective monitoring tools – such as pharmacy prescription tracking, pill counts and electronic drug monitors (EDMs) – exist but are rarely implemented in practice due to cost and time constraints. For the use of condoms, self-reported results may also be biased. We recommend that it may be worth researching how to more effectively evaluate the relevant results in the future.

The US Preventive Services Task Force (44) highlighted that high adherence to PrEP is vital for effectively reducing rates of acquiring HIV. Our research suggested the positive significance of mHealth in promoting users’ good adherence. Current interventions predominantly incorporate the Information-Motivation-Behavioral Skills (IMB) model to enhance adherence optimization through amplification of individual motivation (which is one of the three core areas of the HIV prevention cascade) to engage with PrEP and HIV prevention services (24, 25, 28, 34). At the same time, personalized online support and feedback are provided to adapt to individual daily behavior patterns, which increases user engagement and satisfaction (45, 46). However, these advancements face limitations: (1) Geographic disparities in prevention service accessibility. Although remote intervention increases users’ opportunities to access prevention services and knowledge, PrEP prevention services are limited in resource scarce areas; (2) Digital infrastructure deficits. Rural communities are systematically excluded from online HIV services due to unreliable Internet connections. (3) Privacy and data security risks. Some online intervention platform may have insufficient data protection mechanisms, such as the storage and sharing of sensitive information; the lack of transparency in “which data are obtained” may undermine the credibility of the intervention and the continuous participation of users (47–49). Research shows that some online platforms that provide anonymous support and resources (50) reduce the psychological barriers to seeking help by enabling users to obtain professional advice and emotional support without having their identity. This anonymity reduces the fear of seeking help due to concerns about shame or privacy, thereby increasing the willingness to adopt preventive methods.

We found that mHealth intervention had a positive effect on HIV testing. This might be due to the continuous intervention to improve the awareness of HIV testing and self-management abilities among key populations through educational outreach (51, 52). In addition, certain mobile applications had built-in map features that gathered and showed the locations of HIV testing sites, which helped to alleviate the barrier of geographical accessibility (30). It was worth noting that a considerable part of the studies we included were conducted in the form of self-examination, which had a great relationship with its convenience and privacy. Online platforms offered accessible channels to purchase or request self-test kits, coupled with streamlined user-friendly procedures, which promotes the popularity of self-test (29, 30, 38). Biello’s research obtained similar results with ours (52). The HIV testing kit provided by the mobile app showed the potential to increase the HIV testing rate of men who have sex with men. It found that most participants considered self-collection kits easily accessible and user-friendly through mobile apps. Crucially, they preferred self-collection over physician-administered tests due to the privacy advantages. MacGowan found that participants using self-testing kits had an average testing frequency of 5.2 times in the past 12 months, which was significantly higher than the 1.5 times observed in the control group (53). Currently, an AI-powered mobile chatbot is under investigation, which provides real-time interactive guidance and counseling to offer HIVST services and health education to users (54). Future mHealth interventions need to pay more attention to increasing the coverage of testing services and promoting HIVST because HIV testing has become an important cornerstone of effective prevention strategies and a bridge to help HIV positive individuals enter the HIV treatment cascade as soon as possible (55).

The effectiveness of mHealth interventions in reducing the number of condomless sex events remains unclear. Bruns’ systematic review suggests that SMS interventions can increase the using of sexual health services, but the impact on sexual behavior could not be assessed (56). This may be related to the fact that few studies have reported primary outcomes associated with a reduction in risky sexual behavior. Our findings were not shown to be significant and it is speculated that this may be due to the fact that mHealth interventions have limitations in messaging and are too simplistic in content to allow for sufficiently in-depth guided behaviors. Further high-quality studies are needed in the future to evaluate this outcome.

## 5 Comparison with prior work

Few studies have thoroughly assessed the outcomes of mHealth interventions on promoting good PrEP adherence. Due to the limited availability of relevant studies, most reviews have summarized existing research. Ronen’s study reviewed various digital health platforms in HIV prevention, suggesting their unique potential to influence health behaviors, but the evidence is limited (57). Cao summarized a number of studies and concluded that mHealth interventions offer useful STI and HIV services, with favorable impacts on PrEP interventions (58). A meta-analysis of gamified interventions explored their effects on PrEP adherence and condomless sex (CAS) (18), finding that while gamified interventions did not significantly impact PrEP adherence, but reduce the incidence of condomless sex. The results of this study provide some evidence that mHealth interventions can promote good adherence and achieve better prevention outcomes among people at risk of acquiring HIV. Additional randomized controlled trials are required to confirm the generalizability of our results in diverse populations and settings.

## 6 Limitations

There are some limitations to this paper. Our sample predominantly consisted of individuals aged 18–29 years and the impact of the intervention on older age groups remains unknown. Most studies focused on male participants, with limited representation of women across the evidence base. The majority of research was conducted in the United States, potentially limiting global generalizability due to geographic bias. Variations in mHealth interventions, including their forms, theoretical foundations and delivery methods, may have influenced the evidence outcomes. Due to the nature of the interventions, it is not yet possible to eliminate some risk of bias. The significant heterogeneity observed across included studies may introduce instability to the pooled estimates. The inability to identify specific sources of this variation, potentially stemming from unmeasured methodological or contextual differences, precludes definitive conclusions about the robustness of the statistically significant findings. Future trials should adopt a more rigorous approach, particularly in terms of reducing allocation concealment, intervention implementation and outcome assessment bias. Limitations exist due to the small size of the studies, unexplained heterogeneity in some outcome indicators and insufficient data for in-depth analyses. The quality of evidence for this review was graded as moderate, low or very low with incomplete conclusions that need to be interpreted with caution. Additional studies are needed to further confirm our results.

Currently, long-acting injectable PrEP (LAI-PrEP) has been approved by the US Food and Drug Administration in 2021 (59). Compared to oral PrEP, LAI-PrEP has enormous potential in the field of HIV prevention due to its convenience, confidentiality and other advantages. Research shows that there is a great willingness among men who have sex with men, person who uses drugs and some women to use LAI-PrEP in the future (60–62). At present, there is relatively little research on integrating the mHealth platform with LAI-PrEP. In the future, attention can be paid to remote efficacy testing through dynamic drug metabolism data and a series of technologies such as intelligent reminder systems can be combined to achieve efficient compliance management.

## 7 Conclusion

This study indicates that mHealth technology has a significant impact on the formation of good PrEP compliance in key populations and to some extent optimizes the “effective use” stage of HIV prevention cascade.

## Data Availability

The original contributions presented in the study are included in the article/Supplementary material, further inquiries can be directed to the corresponding author.

## References

[1] UNAIDS. UNAIDS data 2023. (2023). Available online at: https://www.unaids.org/sites/default/files/media_asset/data-book-2023_en.pdf. (Accessed February 11, 2025).

[2] UNAIDS. 2024 global AIDS report—The urgency of now: AIDS at a crossroads. (2024). Available online at: https://www.unaids.org/sites/default/files/media_asset/2024-unaids-global-aids-update_en.pdf. (Accessed February 11, 2025).

[3] Dumchev K Sazonova Y Smyrnov P Cheshun O Pashchuk O Saliuk T . Operationalizing the HIV prevention cascade for PWID using the integrated bio-behavioural survey data from Ukraine. J Int AIDS Soc. (2020) 23:e25509. doi: 10.1002/jia2.25509, PMID: 32602659 PMC7325510

[4] Hargreaves JR Delany-Moretlwe S Hallett TB Johnson S Kapiga S Bhattacharjee P . The HIV prevention cascade: integrating theories of epidemiological, behavioural, and social science into programme design and monitoring. Lancet HIV. (2016) 3:e318–22. doi: 10.1016/S2352-3018(16)30063-7, PMID: 27365206

[5] Schaefer R Gregson S Fearon E Hensen B Hallett TB Hargreaves JR. HIV prevention cascades: a unifying framework to replicate the successes of treatment cascades. Lancet HIV. (2016) 6:e60–6. doi: 10.1016/S2352-3018(18)30327-8 PMC7025885 32066995

[6] Katz DA Golden MR Hughes JP Farquhar C Stekler JD. HIV self-testing increases HIV testing frequency in high-risk men who have sex with men: a randomized controlled trial. J Acquir Immune Defic Syndr. (2018) 78:505–12. doi: 10.1097/QAI.0000000000001709, PMID: 29697595 PMC6037557

[7] Zhu YY Ye ZH Chu ZX Liu Y Wei J Jia L . Effects of HIV self-testing on testing promotion and risk behavior reduction among transgender women in China: randomized controlled trial. J Med Internet Res. (2024) 26:e58591. doi: 10.2196/58591, PMID: 39471367 PMC11558219

[8] Garnett GP Hallett TB Takaruza A Hargreaves J Rhead R Warren M . Providing a conceptual framework for HIV prevention cascades and assessing feasibility of empirical measurement with data from East Zimbabwe: a case study. Lancet HIV. (2016) 3:e297–306. doi: 10.1016/S2352-3018(16)30039-X, PMID: 27365204 PMC4935672

[9] Chen S Fang Y Chan PS Kawuki J Mo P Wang Z. Counseling supporting HIV self-testing and linkage to care among men who have sex with men: systematic review and Meta-analysis. JMIR Public Health Surveill. (2024) 10:e45647. doi: 10.2196/45647, PMID: 38265866 PMC10851126

[10] Zhang J Li C Xu J Hu Z Rutstein SE Tucker JD . Discontinuation, suboptimal adherence, and reinitiation of oral HIV pre-exposure prophylaxis: a global systematic review and meta-analysis. Lancet HIV. (2022) 9:e254–68. doi: 10.1016/S2352-3018(22)00030-3, PMID: 35364026 PMC9124596

[11] Razzaq A Raynes-Greenow C Alam A. Barriers to uptaking HIV testing among pregnant women attending antenatal clinics in low- and middle-income countries: a systematic review of qualitative findings. Aust N Z J Obstet Gynaecol. (2021) 61:817–29. doi: 10.1111/ajo.13430, PMID: 34611883

[12] Lowery C. What is digital health and what do I need to know about it? Obstet Gynecol Clin N Am. (2020) 47:215–25. doi: 10.1016/j.ogc.2020.02.011, PMID: 32451013

[13] Smith B Magnani JW. New technologies, new disparities: the intersection of electronic health and digital health literacy. Int J Cardiol. (2019) 292:280–2. doi: 10.1016/j.ijcard.2019.05.066, PMID: 31171391 PMC6660987

[14] Sun L Qu M Chen B Li C Fan H Zhao Y. Effectiveness of mHealth on adherence to antiretroviral therapy in patients living with HIV: Meta-analysis of randomized controlled trials. JMIR Mhealth Uhealth. (2023) 11:e42799. doi: 10.2196/42799, PMID: 36689267 PMC9903184

[15] Saragih ID Tonapa SI Osingada CP Porta CM Lee BO. Effects of telehealth-assisted interventions among people living with HIV/AIDS: a systematic review and meta-analysis of randomized controlled studies. J Telemed Telecare. (2024) 30:438–50. doi: 10.1177/1357633X211070726, PMID: 34967240

[16] Chandler R Hull S Ross H Guillaume D Paul S Dera N . The pre-exposure prophylaxis (PrEP) consciousness of black college women and the perceived hesitancy of public health institutions to curtail HIV in black women. BMC Public Health. (2020) 20:1172. doi: 10.1186/s12889-020-09248-6, PMID: 32723313 PMC7385954

[17] Shanks S Morelli A Ardines E Holdsworth G Baraitser P. Two-way text messaging to support self-care and delivery of an online sexual health service: mixed methods evaluation. JMIR Mhealth Uhealth. (2020) 8:e17191. doi: 10.2196/17191, PMID: 32815820 PMC7471885

[18] Luo Q Zhang Y Wang W Cui T Li T. mHealth-based gamification interventions among men who have sex with men in the HIV prevention and care continuum: systematic review and Meta-analysis. JMIR Mhealth Uhealth. (2024) 12:e49509. doi: 10.2196/49509, PMID: 38623733 PMC11034423

[19] Patel P Kerzner M Reed JB Sullivan PS El-Sadr WM. Public health implications of adapting HIV pre-exposure prophylaxis programs for virtual service delivery in the context of the COVID-19 pandemic: systematic review. JMIR Public Health Surveill. (2022) 8:e37479. doi: 10.2196/37479, PMID: 35486813 PMC9177169

[20] Page MJ McKenzie JE Bossuyt PM Boutron I Hoffmann TC Mulrow CD . The PRISMA 2020 statement: an updated guideline for reporting systematic reviews. BMJ Clin Res Ed. (2021) 372:n71. doi: 10.1136/bmj.n71 PMC8005924 33782057

[21] Cumpston M Li T Page MJ Chandler J Welch VA Higgins JP . Updated guidance for trusted systematic reviews: a new edition of the Cochrane handbook for systematic reviews of interventions. Cochrane Database Syst Rev. (2019) 10:ED000142. doi: 10.1002/14651858.ED000142, PMID: 31643080 PMC10284251

[22] Evidence Prime Inc. GRADEpro GDT (guideline development tool). (2024). Available online at: https://www.gradepro.org/. (Accessed June 21, 2024).

[23] R Core Team. R: A language and environment for statistical computing. R Foundation for Statistical Computing, Vienna, Austria. (2024). Available online at: https://www.R-project.org/.

[24] Moore DJ Jain S Dubé MP Daar ES Sun X Young J . Randomized controlled trial of daily text messages to support adherence to Preexposure prophylaxis in individuals at risk for human immunodeficiency virus: the TAPIR study. Clin Infect Dis. (2018) 66:1566–72. doi: 10.1093/cid/cix1055, PMID: 29228144 PMC6248545

[25] Liu AY Vittinghoff E von Felten P Rivet Amico K Anderson PL Lester R . Randomized controlled trial of a Mobile health intervention to promote retention and adherence to Preexposure prophylaxis among Young people at risk for human immunodeficiency virus: the EPIC study. Clin Infect Dis. (2019) 68:2010–7. doi: 10.1093/cid/ciy810, PMID: 30239620 PMC6541706

[26] Songtaweesin WN Kawichai S Phanuphak N Cressey TR Wongharn P Saisaengjan C . Youth-friendly services and a mobile phone application to promote adherence to pre-exposure prophylaxis among adolescent men who have sex with men and transgender women at-risk for HIV in Thailand: a randomized control trial. J Int AIDS Soc. (2020) 23:e25564. doi: 10.1002/jia2.25564, PMID: 32869511 PMC7459171

[27] Whiteley L Craker L Haubrick KK Arnold T Mena L Olsen E . The impact of a Mobile gaming intervention to increase adherence to pre-exposure prophylaxis. AIDS Behav. (2021) 25:1884–9. doi: 10.1007/s10461-020-03118-3, PMID: 33483897 PMC8085097

[28] Horvath KJ Helm JL Black A Chase GE Ma J Klaphake J . A pilot randomized controlled trial of an mHealth intervention to improve PrEP adherence among Young sexual minority men. AIDS Behav. (2024) 28:2804–20. doi: 10.1007/s10461-024-04374-3, PMID: 38816592

[29] Zhu X Zhang W Operario D Zhao Y Shi A Zhang Z . Effects of a mobile health intervention to promote HIV self-testing with MSM in China: a randomized controlled trial. AIDS Behav. (2019) 23:3129–39. doi: 10.1007/s10461-019-02452-5, PMID: 30852728 PMC6733671

[30] Biello KB Mayer KH Scott H Valente PK Hill-Rorie J Buchbinder S . The effects of MyChoices and LYNX mobile apps on HIV testing and pre-exposure prophylaxis use by young US sexual minority men: results from a national randomized controlled trial. JMIR Public Health Surveill. (2025) 11:e63428. doi: 10.2196/63428, PMID: 39908084 PMC11840373

[31] Sullivan PS Stephenson R Hirshfield S Mehta CC Zahn R Bauermeister JA . Behavioral efficacy of a sexual health Mobile app for men who have sex with men: randomized controlled trial of Mobile messaging for men. J Med Internet Res. (2022) 24:e34574. doi: 10.2196/34574, PMID: 35025755 PMC8851328

[32] Schnall R Kuhns LM Pearson C Batey DS Bruce J Hidalgo MA . Efficacy of MyPEEPS Mobile, an HIV prevention intervention using Mobile technology, on reducing sexual risk among same-sex attracted adolescent males: a randomized clinical trial. JAMA Netw Open. (2022) 5:e2231853. doi: 10.1001/jamanetworkopen.2022.31853, PMID: 36129712 PMC9494195

[33] Bauermeister JA Tingler RC Demers M Connochie D Gillard G Shaver J . Acceptability and preliminary efficacy of an online HIV prevention intervention for single Young men who have sex with men seeking partners online: the myDEx project. AIDS Behav. (2019) 23:3064–77. doi: 10.1007/s10461-019-02426-7, PMID: 30762190 PMC6693988

[34] Haberer JE Bukusi EA Mugo NR Pyra M Kiptinness C Oware K . Effect of SMS reminders on PrEP adherence in young Kenyan women (MPYA study): a randomised controlled trial. Lancet HIV. (2021) 8:e130–7. doi: 10.1016/S2352-3018(20)30307-6, PMID: 33662265 PMC8289198

[35] Wray TB Chan PA Guigayoma JP Kahler CW. Game plan-a brief web-based intervention to improve uptake and use of HIV pre-exposure prophylaxis (PrEP) and reduce alcohol use among gay and bisexual men: content analysis. JMIR Format Res. (2022) 6:e30408. doi: 10.2196/30408, PMID: 34989679 PMC8771347

[36] Njuguna N Ngure K Mugo N Sambu C Sianyo C Gakuo S . The effect of human immunodeficiency virus prevention and reproductive health text messages on human immunodeficiency virus testing among young women in rural Kenya: a pilot study. Sex Transm Dis. (2019) 43:353–9. doi: 10.1097/OLQ.0000000000000450, PMID: 27200519 PMC4874231

[37] Govender K Beckett S Masebo W Braga C Zambezi P Manhique M . Effects of a short message service (SMS) intervention on reduction of HIV risk behaviours and improving HIV testing rates among populations located near roadside wellness clinics: a cluster randomised controlled trial in South Africa, Zimbabwe and Mozambique. AIDS Behav. (2019) 23:3119–28. doi: 10.1007/s10461-019-02427-6, PMID: 30771133

[38] Lin Y Ren C Liao M Kang D Li C Jiao K . Digital, crowdsourced, multilevel intervention to promote HIV testing among men who have sex with men: cluster randomized controlled trial. J Med Internet Res. (2023) 25:e46890. doi: 10.2196/46890, PMID: 37902831 PMC10644183

[39] Erenrich RK Braun RA Torres-Mendoza DM Stevenson OL Doan TP Klausner JD. Effectiveness of preptech: findings from a 180-day randomized controlled trial of a pre-exposure prophylaxis telehealth intervention. J Acquir Immune Defic Syndr. (2024) 95:463–9. doi: 10.1097/QAI.0000000000003375, PMID: 38133600 PMC10927298

[40] Pustejovsky JE Rodgers MA. Testing for funnel plot asymmetry of standardized mean differences. Res Synth Methods. (2019) 10:57–71. doi: 10.1002/jrsm.1332, PMID: 30506832

[41] Hunter JP Saratzis A Sutton AJ Boucher RH Sayers RD Bown MJ. In meta-analyses of proportion studies, funnel plots were found to be an inaccurate method of assessing publication bias. J Clin Epidemiol. (2014) 67:897–903. doi: 10.1016/j.jclinepi.2014.03.003, PMID: 24794697

[42] Hannaford A Arens Y Koenig H. Real-time monitoring and point-of-care testing: a review of the current landscape of PrEP adherence monitoring. Patient Prefer Adherence. (2021) 15:259–69. doi: 10.2147/PPA.S248696, PMID: 33574659 PMC7873020

[43] Zimmerman RS Mehrotra P Madden T Paul R. The value of assessing self-reported and biological indicators of outcomes in evaluating HIV programs. Curr HIV AIDS Rep. (2021) 18:365–76. doi: 10.1007/s11904-021-00560-3 33993397

[44] US Preventive Services Task Force Owens DK Davidson KW Krist AH Barry MJ Cabana M . Preexposure prophylaxis for the prevention of HIV infection: US preventive services task force recommendation statement. JAMA. (2019) 321:2203–13. doi: 10.1001/jama.2019.6390, PMID: 31184747

[45] Zemlak JL Singer R Christianson J Stenersen M Singh M Lerret S. Telehealth resources and utilization interest among women who sell sex: an explanatory sequential mixed methods study. Public Health Pract. (2024) 7:100502. doi: 10.1016/j.puhip.2024.100502, PMID: 38800541 PMC11127207

[46] Pintye J Rogers Z Kinuthia J Mugwanya KK Abuna F Lagat H . Two-way short message service (SMS) communication may increase pre-exposure prophylaxis continuation and adherence among pregnant and postpartum women in Kenya. Glob Health Sci Pract. (2020) 8:55–67. doi: 10.9745/GHSP-D-19-00347, PMID: 32139420 PMC7108943

[47] Owens C Buchanan E Fisher CB. Perceived risks and benefits of tele PrEP interventions: an interview study with rural sexual minority men in Texas. J Rural Health. (2024) 41:2886. doi: 10.1111/jrh.12886, PMID: 39367578

[48] Hubach RD O’Neil AM Stowe M Hamrick J Giano Z Currin JM. Preferred methods of HIV and sexually transmissible infection screening delivery among a rural sample of men who have sex with men. AIDS Patient Care STDs. (2020) 34:470–6. doi: 10.1089/apc.2020.0170, PMID: 33147083

[49] Jones J Edwards OW Merrill L Sullivan PS Stephenson R. Interest in HIV prevention Mobile phone apps: focus group study with sexual and gender minority persons living in the rural southern United States. JMIR Format Res. (2022) 6:e38075. doi: 10.2196/38075, PMID: 35699980 PMC9237777

[50] Giovenco D Muessig KE Horvitz C Biello KB Liu AY Horvath KJ . Adapting technology-based HIV prevention and care interventions for youth: lessons learned across five U.S. adolescent trials network studies. mHealth. (2021) 7:21. doi: 10.21037/mhealth-20-43, PMID: 33898590 PMC8063021

[51] Ko JS Stafylis C Klausner JD. Mobile health promotion of human immunodeficiency virus self-testing in the United States. mHealth. (2020) 6:10. doi: 10.21037/mhealth.2019.10.05, PMID: 32190621 PMC7063269

[52] Biello KB Horvitz C Mullin S Mayer KH Scott H Coleman K . HIV self-testing and STI self-collection via mobile apps: experiences from two pilot randomized controlled trials of young men who have sex with men. mHealth. (2021) 7:26. doi: 10.21037/mhealth-20-70, PMID: 33898595 PMC8063023

[53] MacGowan RJ Chavez PR Borkowf CB Owen SM Purcell DW Mermin JH . Effect of internet-distributed HIV self-tests on HIV diagnosis and behavioral outcomes in men who have sex with men: a randomized clinical trial. JAMA Intern Med. (2020) 180:117–25. doi: 10.1001/jamainternmed.2019.5222, PMID: 31738378 PMC6865312

[54] Chen S Zhang Q Chan CK Yu FY Chidgey A Fang Y . Evaluating an innovative HIV self-testing service with web-based, real-time counseling provided by an artificial intelligence Chatbot (HIVST-Chatbot) in increasing HIV self-testing use among Chinese men who have sex with men: protocol for a noninferiority randomized controlled trial. JMIR Res Protocols. (2023) 12:e48447. doi: 10.2196/48447, PMID: 37389935 PMC10365592

[55] Wray T Chan PA Simpanen E Operario D. eTEST: developing a smart home HIV testing kit that enables active, real-time follow-up and referral after testing. JMIR Mhealth Uhealth. (2017) 5:e62. doi: 10.2196/mhealth.6491, PMID: 28483744 PMC5440737

[56] Burns K Keating P Free C. A systematic review of randomised control trials of sexual health interventions delivered by mobile technologies. BMC Public Health. (2016) 16:778. doi: 10.1186/s12889-016-3408-z, PMID: 27514851 PMC4982424

[57] Ronen K Grant E Copley C Batista T Guthrie BL. Peer group focused eHealth strategies to promote HIV prevention, testing, and care engagement. Curr HIV/AIDS Rep. (2020) 17:557–76. doi: 10.1007/s11904-020-00527-w, PMID: 32794071 PMC7492479

[58] Cao B Bao H Oppong E Feng S Smith KM Tucker JD . Digital health for sexually transmitted infection and HIV services: a global scoping review. Curr Opin Infect Dis. (2020) 33:44–50. doi: 10.1097/QCO.0000000000000619, PMID: 31789695 PMC7152691

[59] Rivera CG Zeuli JD Smith BL Johnson TM Bhatia R Otto AO . HIV pre-exposure prophylaxis: new and upcoming drugs to address the HIV epidemic. Drugs. (2023) 83:1677–98. doi: 10.1007/s40265-023-01963-9, PMID: 38079092

[60] Philbin MM Parish C Kinnard EN Reed SE Kerrigan D Alcaide ML . Interest in long-acting injectable pre-exposure prophylaxis (LAI PrEP) among women in the women’s interagency HIV study (WIHS): a qualitative study across six cities in the United States. AIDS Behav. (2021) 25:667–78. doi: 10.1007/s10461-020-03023-9, PMID: 32910351 PMC7886938

[61] Fu J Dai Z Wang H Si M Chen X Wu Y . Willingness to use long-acting injectable PrEP among HIV-negative/unknown men who have sex with men in mainland China: a cross-sectional online survey. PLoS One. (2023) 18:e0293297. doi: 10.1371/journal.pone.0293297, PMID: 37856527 PMC10586652

[62] Shrestha R DiDomizio EE Kim RS Altice FL Wickersham JA Copenhaver MM. Awareness about and willingness to use long-acting injectable pre-exposure prophylaxis (LAI-PrEP) among people who use drugs. J Subst Abus Treat. (2020) 117:108058. doi: 10.1016/j.jsat.2020.108058, PMID: 32811633 PMC7438607

[63] Grant RM Lama JR Anderson PL McMahan V Liu AY Vargas L . Preexposure chemoprophylaxis for HIV prevention in men who have sex with men. N Engl J Med. (2010) 363:2587–99. doi: 10.1056/NEJMoa1011205, PMID: 21091279 PMC3079639

