# Supplementary material for: Impact of mHealth on enhancing pre-exposure prophylaxis adherence and strengthening the HIV prevention cascade among key populations: a systematic review and meta-analysis
Source: Front Public Health. 2025 Jun 26;13:1600773. doi: 10.3389/fpubh.2025.1600773 (PMC12240955; doi:10.3389/fpubh.2025.1600773)
Supplement: Supplementary file 1 [file Supplementary_file_1.docx]

| **Databases** | **Strategies** | **Results** |
| --- | --- | --- |
| **PubMed** | #1 HIV[MeSH Terms]  Sort by: Most Recent | 110595 |
|  | #2 HIV Infections[MeSH Terms]  Sort by: Most Recent | 329768 |
|  | #3 (HIV[Text Word]) OR (HIV/AIDS[Text Word]) OR (human immunodeficiency virus[Text Word]) OR (human immuno-deficiency virus[Text Word]) OR (AIDS[Text Word]) OR (Acquired Immunodeficiency Syndrome[MeSH Terms]) OR (acquired immuno-deficiency syndrome[Text Word]) OR (acquired immune-deficiency syndrome[Text Word])  Sort by: Most Recent | 550407 |
|  | #4 hiv‐1*[Text Word] OR hiv‐2*[Text Word] OR hiv1[Text Word] OR hiv2[Text Word] OR HIV infect*[Text Word] OR human immunedeficiency virus[Text Word] OR human immune‐deficiency virus[Text Word] OR ((human immun*) AND (deficiency virus[Text Word])) OR acquired immunodeficiency syndrome[Text Word] OR acquired immunedeficiency syndrome[Text Word] OR acquired immuno‐deficiency syndrome[Text Word] OR acquired immune‐deficiency syndrome[Text Word] OR ((acquired immun*) AND (deficiency syndrome[Text Word])) OR "sexually transmitted diseases, viral"[MESH:NoExp]  Sort by: Most Recent | 389236 |
|  | #5 Telemedicine[MeSH Terms]  Sort by: Most Recent | 52443 |
|  | #6 Mobile Health[MeSH Terms]  Sort by: Most Recent | 52443 |
|  | #7 Health, Mobile[MeSH Terms]  Sort by: Most Recent | 52443 |
|  | #8 mHealth[MeSH Terms]  Sort by: Most Recent | 52443 |
|  | #9 Telehealth[MeSH Terms]  Sort by: Most Recent | 52443 |
|  | #10 eHealth[MeSH Terms]  Sort by: Most Recent | 52443 |
|  | #11 Internet-Based Intervention[MeSH Terms]  Sort by: Most Recent | 1638 |
|  | #12 Internet Based Intervention[MeSH Terms]  Sort by: Most Recent | 1638 |
|  | #13 Internet-Based Interventions[MeSH Terms]  Sort by: Most Recent | 1638 |
|  | #14 Online Intervention[MeSH Terms]  Sort by: Most Recent | 1638 |
|  | #15 Mobile Application[MeSH Terms]  Sort by: Most Recent | 14768 |
|  | #16 Mobile App[MeSH Terms]  Sort by: Most Recent | 14768 |
|  | #17 (smart phone[Text Word]) OR (phone[Text Word]) OR (mobile phone[Text Word]) OR (blog[Text Word]) OR (social media[Text Word]) OR (social networking[Text Word]) OR (mobile technology[Text Word]) OR (telehealth[Text Word]) OR (tele-health[Text Word]) OR (video[Text Word]) OR (computer[Text Word]) OR (online platform[Text Word])  Sort by: Most Recent | 1154550 |
|  | #18 Wireless Technology[MeSH Terms]  Sort by: Most Recent | 5191 |
|  | #19 Wireless Communication[Mesh]  Sort by: Most Recent | 774 |
|  | #20 Wearable Electronic Devices[Mesh]  Sort by: Most Recent | 23188 |
|  | #21 wireless medical devices[Text Word] OR wireless health monitoring[Text Word] OR Bluetooth[tiab] OR BLE[tiab] OR Wireless Network[tiab]  Sort by: Most Recent | 3746 |
|  | #22 Pre-Exposure Prophylaxis[Mesh]  Sort by: Most Recent | 6050 |
|  | #23 pre‐exposure prophylaxis[Tiab] OR preexposure prophylaxis[Tiab] OR PREP[Tiab] OR anti‐retroviral chemoprophylaxis[Tiab] OR antiretroviral chemoprophylaxis[Tiab] OR chemoprevention[MeSH Terms] OR chemoprevention[Tiab] OR HIV prophylaxis[Tiab]  Sort by: Most Recent | 47709 |
|  | #24 tenofovir[Text Word] OR TNF[Text Word] OR TDF[Text Word] OR PMPA[Text Word] OR viread[Text Word] OR emtricitabine[Text Word] OR EMC[Text Word] OR truvada[Text Word] OR emtriva[Text Word] OR coviracil[Text Word]  Sort by: Most Recent | 260182 |
|  | #25 ("Condoms"[Mesh] OR "condom use"[tiab] OR "safe sex"[tiab] OR "barrier method"[tiab]) OR ('HIV self-testing'/exp OR 'HIV selftest':ti,ab OR 'home test kit':ti,ab OR 'HIV test' OR 'HIV prevention' OR ("HIV Infections/prevention and control"[Mesh] OR "HIV prevention"[tiab]))  Sort by: Most Recent | 181651 |
|  | #26 Awareness[Mesh]  Sort by: Most Recent | 22918 |
|  | #27 Recognition, Psychology[Mesh]  Sort by: Most Recent | 22907 |
|  | #28 Comprehension[Mesh]  Sort by: Most Recent | 18709 |
|  | #29 Knowledge[Mesh]  Sort by: Most Recent | 15527 |
|  | #30 Health Knowledge, Attitudes, Practice[Mesh]  Sort by: Most Recent | 136547 |
|  | #31 Health Education[Mesh]  Sort by: Most Recent | 272698 |
|  | #32 (Awareness[Text Word]) OR (Knowledge[Text Word]) OR (Understanding[Text Word]) OR (Familiarity[Text Word]) OR (Comprehension[Text Word]) OR (Education[Text Word])  Sort by: Most Recent | 3463672 |
|  | #33 Patient Compliance[Mesh]  Sort by: Most Recent | 153788 |
|  | #34 Medication Adherence[Mesh]  Sort by: Most Recent | 93122 |
|  | #35 Health Behavior[Mesh]  Sort by: Most Recent | 441014 |
|  | #36 (medication adherence[Text Word]) OR (drug Compliance [Text Word]) OR (treatment Compliance[Text Word]) OR (medication conformity[Text Word]) OR (drug adherence[Text Word])  Sort by: Most Recent | 44922 |
|  | #37 (randomized controlled trial [Publication Type] OR controlled clinical trial [Publication Type] OR randomized [Tiab] OR placebo [Tiab] OR drug therapy [sh] OR randomly [Tiab] OR trial [Tiab] OR groups [Tiab]) NOT (animals [MeSH Terms] NOT humans [MeSH Terms])  Sort by: Most Recent | 5596113 |
|  | #38 #1 OR #2 OR #3 OR #4  Sort by: Most Recent | 553869 |
|  | #39 #5 OR #6 OR #7 OR #8 OR #9 OR #10 OR #11 OR #12 OR #13 OR #14 OR #15 OR #16 OR #17 OR #18 OR #19 OR #20 OR #21  Sort by: Most Recent | 1216517 |
|  | #40 #22 OR #23 OR #24 OR#25  Sort by: Most Recent | 476478 |
|  | #40 #26 OR #27 OR #28 OR #29 OR #30 OR #31 OR #32 OR #33 OR #34 OR #35 OR #36  Sort by: Most Recent | 3821222 |
|  | #41 #37 AND #38 AND #39 AND #40 AND #41  Sort by: Most Recent | 1174 |
| **Cochrane** | #1 (HIV OR “HIVAIDS” OR Human Immunodeficiency Virus* OR AIDS OR Acquired Immunodeficiency Syndrome* OR Acquired Immunologic Deficiency Syndrome* OR Acquired Immun* Deficiency Syndrome* OR “HIV 1” OR “HIV 2” OR HIV I OR HIV II):ti,ab,kw(Word variations have been searched) | 40288 |
|  | #2 MeSH descriptor: [HIV Infections] explode all trees | 17088 |
|  | #3 MeSH descriptor: [HIV] explode all trees | 4127 |
|  | #4 #1 OR #2 OR #3 in Trials | 40288 |
|  | #5 MeSH descriptor: [Telemedicine] explode all trees | 5312 |
|  | #6 MeSH descriptor: [Internet-Based Intervention] explode all trees | 896 |
|  | #7 (Mobile Health OR mHealth OR Telehealth OR eHealth OR Online Intervention):ti,ab,kw(Word variations have been searched) | 38464 |
|  | #8 (Mobile Application OR Mobile App OR APP OR telehealthcare OR phone):ti,ab,kw(Word variations have been searched) | 35606 |
|  | #9 (blog OR social media OR social networking OR mobile technology OR video):ti,ab,kw(Word variations have been searched) | 40669 |
|  | #10 (Computer OR online platform OR WeChat):ti,ab,kw(Word variations have been searched) | 65136 |
|  | #11 MeSH descriptor: [Wireless Technology] explode all trees | 74 |
|  | #12 (wireless technolog* OR wireless communication* OR remote sensing OR data transmission):ti,ab,kw(Word variations have been searched) | 14861 |
|  | #13 #5 OR #6 OR #7 OR #8 OR #9 OR #10 OR #11 OR #12 in Trials | 157186 |
|  | #14 MeSH descriptor: [Pre-Exposure Prophylaxis] explode all trees | 539 |
|  | #15 (preexposure prophylaxis OR PrEp OR anti‐retroviral chemoprophylaxis OR antiretroviral chemoprophylaxis OR chemoprevention OR HIV prophylaxis) :ti,ab,kw(Word variations have been searched) | 6318 |
|  | #16(Tenofovir OR TNF OR TDF OR PMPA OR viread OR emtricitabine OR EMC OR truvada OR emtriva OR coviracil) :ti,ab,kw(Word variations have been searched) | 22760 |
|  | #17 MeSH descriptor: [condom] explode all trees | 830 |
|  | #18 MeSH descriptor: [Self-Testing] explode all trees | 1710 |
|  | #19 (condom adherence OR consistent condom use OR HIV self-test OR home-based test kit OR HIV test ) :ti,ab,kw(Word variations have been searched) | 17606 |
|  | #20 #14 OR #15 OR #16 OR #17 OR #18 OR #19 in Trials | 44417 |
|  | #21 MeSH descriptor: [Awareness] explode all trees | 1240 |
|  | #22 MeSH descriptor: [Recognition, Psychology] explode all trees | 925 |
|  | #23 MeSH descriptor: [Comprehension] explode all trees | 966 |
|  | #24 MeSH descriptor: [Patient Medication Knowledge] explode all trees | 16 |
|  | #25 MeSH descriptor: [Health Knowledge, Attitudes, Practice] explode all trees | 8434 |
|  | #26 MeSH descriptor: [Health Education] explode all trees | 26247 |
|  | #27 (Cognition OR Understanding OR Knowledge):ti,ab,kw(Word variations have been searched) | 130246 |
|  | #28 MeSH descriptor: [Patient Compliance] explode all trees | 20258 |
|  | #29 MeSH descriptor: [Medication Adherence] explode all trees | 8996 |
|  | #30 MeSH descriptor: [Health Behavior] explode all trees | 51752 |
|  | #31 (drug Compliance OR treatment Compliance OR medication conformity OR drug adherence OR compli* OR adheren*):ti,ab,kw(Word variations have been searched) | 364445 |
|  | #32 # #21 OR #22 OR #23 OR #24 OR #25 OR #26 OR #27 OR #28 OR #29 OR #30 OR #31 in Trials | 500146 |
|  | #33 #4 AND #13 AND #20 AND #32 in Trials | 1508 |
| **Web of science** | #1 ((((((((((((((((((TS=(HIV) OR TS=(HIV Infections)) OR TS=(HIV/AIDS)) OR TS=(human immunodeficiency virus)) OR TS=(human immuno-deficiency virus)) OR TS=(AIDS)) OR TS=(Acquired Immunodeficiency Syndrome)) OR TS=(acquired immuno-deficiency syndrome)) OR TS=(acquired immune-deficiency syndrome)) OR TS=(Acquired Immunologic Deficiency Syndrome)) OR TS=(people living with HIV)) OR TS=(PLWH)) OR TS=(people living with AIDS)) OR TS=(PLWA)) OR TS=(people living with HIV/AIDS)) OR TS=(PLWHA)) OR TS=(HIV 1)) OR TS=(HIV 2)) OR TS=( HIV I)) OR TS=( HIV II) | 2029912 |
|  | #2 (((((((((((((((((TS=(Telemedicine) OR TS=(Mobile Health)) OR TS=(mHealth)) OR TS=(Telehealth)) OR TS=(eHealth)) OR TS=(Internet-Based Intervention)) OR TS=(Online Intervention)) OR TS=(Mobile Application)) OR TS=(Mobile App)) OR TS=(APP)) OR TS=(smart phone)) OR TS=(phone)) OR TS=(mobile phone)) OR TS=(blog)) OR TS=(social media)) OR TS=(social networking)) OR TS=((mobile technology)) OR TS=(video)) OR TS=(computer)) OR TS=(online platform) OR TS=(wireless technolog*) OR TS=(wireless communication*) OR TS=(remote sensing ) | 8721160 |
|  | #3 (((((((((((((((TS=(Pre-Exposure Prophylaxis) OR TS=(preexposure prophylaxis)) OR TS=( PrEp)) OR TS=(antiretroviral chemoprophylaxis)) OR TS=(anti‐retroviral chemoprophylaxis)) OR TS=(chemoprevention)) OR TS=(HIV prophylaxis)) OR TS=(Tenofovir)) OR TS=(TNF)) OR TS=(TDF)) OR TS=(PMPA)) OR TS=(viread)) OR TS=(emtricitabine)) OR TS=(EMC)) OR TS=(truvada)) OR TS=(emtriva)) OR TS=(coviracil) OR TS=(HIV prevention) OR TS=AIDS prevention OR TS=HIV risk reduction OR TS=("condom use" OR "condom adherence" OR "safe sex" OR "barrier method") OR TS=("HIV self-testing" OR "HIV self-test" OR "home-based testing") | 737393 |
|  | #4 (((((((((((((((((TS=(Awareness) OR TS=(Recognition)) OR TS=(Comprehension)) OR TS=(Patient Medication Knowledge)) OR TS=(Health Knowledge)) OR TS=(Health Attitudes)) OR TS=(Health Practice)) OR TS=(Health Education)) OR TS=(Cognition)) OR TS=(Understanding)) OR TS=(Patient Compliance)) OR TS=(Medication Adherence)) OR TS=(Health Behavior)) OR TS=(drug Compliance)) OR TS=(treatment Compliance)) OR TS=(medication conformity)) OR TS=(drug adherence)) OR TS=(compli*)) OR TS=(adheren*) | 15342999 |
|  | #5 TS=(randomized controlled trial) OR TS=(RCT) | 850659 |
|  | #6 #1 AND #2 AND #3 AND #4 AND #5 | 2628 |
| **Embase** | #1 'acquired immune deficiency syndrome'/exp | 594294 |
|  | #2 'human immunodeficiency virus'/exp | 229567 |
|  | #3 hiv:ab,ti OR 'hiv infections':ab,ti OR AIDS:ab,ti OR 'hiv/aids':ab,ti OR 'people living with hiv':ab,ti OR plwh:ab,ti OR 'people living with hiv/aids':ab,ti OR plwha:ab,ti OR 'aids patient':ab,ti | 600558 |
|  | #4 'telehealth'/exp | 106766 |
|  | #5 'web-based intervention'/exp | 4295 |
|  | #6 'telecare'/exp | 2013 |
|  | #7 'telemedicine'/exp | 84839 |
|  | #8 'mobile health':ab,ti OR mhealth:ab,ti OR ehealth:ab,ti OR 'internet-based intervention':ab,ti OR 'online intervention':ab,ti OR 'mobile application':ab,ti OR app:ab,ti OR phone:ab,ti OR blog:ab,ti OR 'social media':ab,ti OR 'mobile technology':ab,ti OR video:ab,ti OR computer:ab,ti OR 'online platform':ab,ti | 710853 |
|  | #9 'wireless technolog*':ab,ti OR 'wireless communication*':ab,ti | 4060 |
|  | #10 'wearable electronic device'/exp | 13937 |
|  | #11 'remote monitoring'/exp | 23155 |
|  | #12 'pre-exposure prophylaxis '/exp | 12234 |
|  | #13 'preexposure prophylaxis':ab,ti OR PrEp:ab,ti OR 'anti‐retroviral chemoprophylaxis':ab,ti OR 'antiretroviral chemoprophylaxis':ab,ti OR chemoprevention:ab,ti OR 'HIV prophylaxis':ab,ti | 35085 |
|  | #14 Tenofovir:ti,ab OR TNF:ti,ab OR TDF:ti,ab OR PMPA:ti,ab OR viread:ti,ab OR emtricitabine:ti,ab OR EMC:ti,ab OR Truvada:ti,ab OR emtriva:ti,ab OR coviracil:ti,ab | 353637 |
|  | #15 (( 'hiv infection'/exp OR 'prevention' OR ('HIV prevention':ti,ab OR 'PrEP':ti,ab)) OR ('condom'/exp OR ('condom use':ti,ab OR 'safe sex':ti,ab)) OR ('self-testing'/exp OR ('HIV self-testing':ti,ab OR 'home-based testing':ti,ab))) | 3485276 |
|  | #16 'education'/exp OR education | 3167326 |
|  | #17 'knowledge'/exp OR knowledge | 1470897 |
|  | #18 'recognition'/exp OR recognition | 630941 |
|  | #19 'comprehension'/exp OR comprehension | 66616 |
|  | #20 'patient compliance'/exp OR 'patient compliance' | 227770 |
|  | #21 'medication compliance'/exp OR 'medication compliance' | 57393 |
|  | #22 'attitude to health'/exp OR 'attitude to health' | 143138 |
|  | #23 'Patient Medication Knowledge':ab,ti OR 'Health Practice':ab,ti OR 'Health Education':ab,ti OR Cognition:ab,ti OR Understanding:ab,ti OR 'Medication Adherence':ab,ti OR 'Health Behavior':ab,ti OR 'drug Compliance':ab,ti OR 'treatment Compliance':ab,ti OR 'medication conformity':ab,ti OR 'drug adherence':ab,ti | 1840276 |
|  | #24 'awareness'/exp OR awareness | 378859 |
|  | #25 compli* OR adheren*  OR educat* | 7481339 |
|  | #26 'randomized controlled trial'/exp | 922705 |
|  | #27 #1 OR #2 OR #3 | 1064497 |
|  | #28 #4 OR #5 OR #6 OR #7 OR #8 OR #9 OR #10 OR #11 | 826044 |
|  | #29 #12 OR #13 OR #14 OR #15 | 3801468 |
|  | #30 #16 OR #17 OR #18 OR #19 OR #20 OR #21 OR #22 OR #23 OR #24 OR#25 | 10851259 |
|  | #31 #26 AND #27 AND #28 AND #29 AND #30 | 788 |
| **OVID** | #1 Acquired Immunodeficiency Syndrome/ | 14309 |
|  | #2 HIV/ or HIV Infections/ or HIV-1/ or HIV-2/ | 98608 |
|  | #3 *Human immunodeficiency virus/ | 36877 |
|  | #4 (acquired immun* and deficiency syndrome).ti,ab. | 3108 |
|  | #5 (HIV or HIV AIDS or human immunodeficiency virus or human immune deficiency virus or human immune-deficiency virus or human immune-deficiency virus or hiv-1* or hiv-2* or hiv1 or hiv2 or acquired immunodeficiency syndromes or acquired immune deficiency syndrome or acquired immune-deficiency syndrome or acquired immuno-deficiency syndrome or Acquired Immunologic Deficiency Syndrome or people living with HIV or PLWH or people living with AIDS or PLWHA).ti,ab. | 300474 |
|  | #6 Telemedicine/ or mHealth/ or Mobile Health/ or Telehealth/ or eHealth/ | 42684 |
|  | #7 Internet-Based Intervention/ or Internet Based Intervention/ or Internet-Based Interventions/ or Online Intervention/ or Mobile Application/ or Mobile App/ | 11767 |
|  | #8 (smart phone or phone or mobile phone or blog or social media or social networking or mobile technology or telehealth or tele-health or video or computer or online platform).ti,ab. | 361509 |
|  | #9 wireless technology/ | 2369 |
|  | #10 wireless medical devices/ OR wireless health monitoring/ | 0 |
|  | #11 Wearable Electronic Devices/ OR Remote Monitoring/ OR wireless communication/ | 7960 |
|  | #12 Pre-Exposure Prophylaxis/ | 6026 |
|  | #13 (preexposure prophylaxis or PrEp or anti-retroviral chemoprophylaxis or antiretroviral chemoprophylaxis or chemoprevention or HIV prophylaxis or Tenofovir or TNF or TDF or PMPA or viread or emtricitabine or EMC or truvada or emtriva or coviracil).ti,ab. | 124148 |
|  | #14 ("HIV prevention" OR "AIDS prevention" OR "HIV risk reduction") OR ("condom use" OR "condom adherence" OR "barrier method") OR ("HIV self-testing" OR "HIV self-test" OR "home-based testing").ti,ab. | 72042 |
|  | #15 Recognition, Psychology/ | 7579 |
|  | #16 Comprehension/ | 17547 |
|  | #17 Patient Medication Knowledge/ | 50462 |
|  | #18 Health Knowledge, Attitudes, Practice/ | 19093 |
|  | #19 Health Education/ | 70118 |
|  | #20 Patient Compliance/ or Medication Adherence/ | 83354 |
|  | #21 Health Behavior/ | 57558 |
|  | #22 (Cognition or Understanding or Knowledge or drug Compliance or treatment Compliance or medication conformity or drug adherence or compli* OR adheren*).ti,ab. | 2645998 |
|  | #23 Awareness/ | 101287 |
|  | #24 Randomized controlled trial/ | 332066 |
|  | #25 Random$.ti,ab. | 1297739 |
|  | #26 Random Allocation/ | 21228 |
|  | #27 #1 OR #2 OR #3 OR #4 OR #5 | 319061 |
|  | #28 #6 OR #7 OR #8 OR #9 OR #10 OR #11 | 403021 |
|  | #29 #12 OR #13 OR #14 | 192273 |
|  | #30 #15 OR #16 OR #17 OR #18 OR #19 OR #20 OR #21 OR #22 OR #23 | 2882372 |
|  | #31 #24 OR #25 OR #26 | 1346930 |
|  | #32 #27 AND #28 AND #29 AND #30 AND #31 | 167 |
| **CINAHL** | S1: TI HIV OR TI AIDS OR TI HIV/AIDS OR TI HIV Infections OR TI human immunodeficiency virus OR TI Acquired Immunodeficiency Syndrome OR TI people living with HIV OR TI PLWH OR TI people living with AIDS OR TI PLWA OR TI HIV/AIDS patient OR TI people living with HIV/AIDS  Expanders - Apply equivalent subjects Search modes - Boolean/Phrase | 20897 |
|  | S2: TI hiv1 OR TI hiv2 OR TI hiv-1 OR hiv-2  Expanders - Apply equivalent subjects Search modes - Boolean/Phrase | 3899 |
|  | S3: TI Telemedicine OR TI Mobile Health OR TI mHealth OR TI Telehealth OR TI eHealth OR TI Internet-Based Intervention OR TI Online Intervention OR TI Mobile Application OR TI Mobile App OR TI APP OR TI smart phone OR TI blog OR TI wireless technology OR TI wireless medical devices OR TI wireless health monitoring OR TI Wearable Electronic Devices OR TI Remote Monitoring OR TI wireless communication  Expanders - Apply equivalent subjects Search modes - Boolean/Phrase | 24325 |
|  | S4: TI Pre-Exposure Prophylaxis OR TI PrEp OR TI anti‐retroviral chemoprophylaxis OR TI chemoprevention OR TI HIV prophylaxis  Expanders - Apply equivalent subjects Search modes - Boolean/Phrase | 4295 |
|  | S5: TI Tenofovir OR TI TNF OR TI TDF OR TI PAMA OR TI viread OR TI emtricitabine OR TI EMC OR TI truvada OR TI emtriva OR TI coviracil  Expanders - Apply equivalent subjects Search modes - Boolean/Phrase | 3556 |
|  | S6: TI HIV prevention OR TI AIDS prevention OR TI condom OR TI Self-Testing OR TI condom use OR TI condom adherence  Expanders - Apply equivalent subjects Search modes - Boolean/Phrase | 4559 |
|  | S7: TI Awareness OR TI Recognition OR TI Comprehension OR TI Patient Medication Knowledge OR TI Health Knowledge OR TI Health Attitudes OR TI Health Practice OR TI Health Education OR TI Cognition OR TI Understanding  Expanders - Apply equivalent subjects Search modes - Boolean/Phrase | 82791 |
|  | S8: TI Patient Compliance OR TI Medication Adherence OR TI Health Behavior OR TI drug Compliance OR TI treatment Compliance OR TI medication conformity OR TI drug adherence OR TI compli* OR TI adheren*  Expanders - Apply equivalent subjects Search modes - Boolean/Phrase | 103507 |
|  | S9: S1 OR S2  Expanders - Apply equivalent subjects Search modes - Boolean/Phrase | 24730 |
|  | S10: S4 OR S5 OR S6  Expanders - Apply equivalent subjects Search modes - Boolean/Phrase | 12327 |
|  | S11: S7 OR S8  Expanders - Apply equivalent subjects Search modes - Boolean/Phrase | 185013 |
|  | S12: S3 AND S9 AND S10 AND S11  Expanders - Apply equivalent subjects Search modes - Boolean/Phrase | 10 |
| **CNKI** | #1 TKA=('人类免疫缺陷病毒' + 'human immuno-deficiency virus' + 'human immunodeficiency virus'+ 'human immunodeficiency viruses' + '人体免疫缺陷病毒' + '获得性免疫缺陷综合症'+ '艾滋病' + '人类获得性免疫缺陷病毒'+ '爱滋病' + '人免疫缺陷病毒' + '获得性免疫缺乏综合征'+ 'AIDS' + 'Acquired Immune Deficiency Syndrome'+ 'HIV') | 97688 |
|  | #2 TKA=('移动医疗' + 'mobile health' + 'mobile telemedicine' + '移动健康' + '移动应用' + '智能手机') OR (TKA=('手机' + '电子' + '数字' + '远程' + '在线' + '无线') AND TKA=('医疗' + '健康' + '干预')) | 148021 |
|  | #3 TKA=('PrEp' + '暴露前预防' + 'pre-exposure prophylaxis' + '暴露前用药' + '暴露前干预' + '暴露前策略' + '感染预防' + '高危暴露' + '药物预防' + '风险预防' + '保护措施' + '自我检测' + '风险检测' + '避孕套' + '安全套') | 69092 |
|  | #4 TKA=('知晓' + '知晓率' + '认知' + '理解' + '知识' + '态度' + '依从性' + '遵从性' + '健康行为') | 3140354 |
|  | #5 TKA=('随机对照' + '随机分配' + '随机分组'+ 'RCT ' + '随机') | 2039789 |
|  | #6 #1 AND #2 AND #3 AND #4 AND #5 | 13 |
