# Supplementary material for: Impact of mHealth on enhancing pre-exposure prophylaxis adherence and strengthening the HIV prevention cascade among key populations: a systematic review and meta-analysis
Source: Front Public Health. 2025 Jun 26;13:1600773. doi: 10.3389/fpubh.2025.1600773 (PMC12240955; doi:10.3389/fpubh.2025.1600773)
Supplement: Supplementary file 2 [file Supplementary_file_2.pdf]

Author(s):
Question:
Setting:
Bibliography:

Certainty assessment							№ of patients		Effect		Certainty	Importance
**№ of studies**	**Study design**	**Risk of bias**	**Inconsistency**	**Indirectness**	**Imprecision**	**Other considerations**			**Relative (95% CI)**	**Absolute (95% CI)**		
**Good adherence to PrEP at 12-week follow-up**												
7[a]	randomised trials	serious[b]	not serious	not serious	not serious	none			**OR 1.59** (1.14 to 2.23)	**2 fewer per 1,000** (from 2 fewer to 1 fewer)	⊕⊕⊕◯ Moderate[b]	CRITICAL
**Good adherence to PrEP at 24-week follow-up**												
7[a]	randomised trials	serious[b]	not serious	not serious	serious[c]	none			**OR 1.60** (1.09 to 2.35)	**2 fewer per 1,000** (from 2 fewer to 1 fewer)	⊕⊕◯◯ Low[b,c]	CRITICAL
**HIV testing at 12-week follow-up**												
5[a]	randomised trials	serious[b]	not serious	not serious	serious[c]	none			**OR 1.19** (0.95 to 1.48)	**1 fewer per 1,000** (from 1 fewer to 1 fewer)	⊕⊕◯◯ Low[b,c]	CRITICAL
**HIV testing at 24-week follow-up**												
7[a]	randomised trials	serious[b]	not serious	not serious	serious[c]	none			**OR 1.63** (1.39 to 1.90)	**2 fewer per 1,000** (from 2 fewer to 1 fewer)	⊕⊕◯◯ Low[b,c]	IMPORTANT
**PrEP use at 12-week follow-up**												
6[a]	randomised trials	serious[b]	serious[d]	not serious	serious[e]	none			**OR 3.38** (1.95 to 5.86)	**3 fewer per 1,000** (from 6 fewer to 2 fewer)	⊕◯◯◯ Very low[b,d,e]	IMPORTANT
**PrEP use at 24-week follow-up**												
6[a]	randomised trials	serious[b]	serious[d]	not serious	serious[e]	none			**OR 1.86** (1.04 to 3.41)	**2 fewer per 1,000** (from 3 fewer to 1 fewer)	⊕◯◯◯ Very low[b,d,e]	IMPORTANT
**Condomless sex events at 12-week follow-up**												
3	randomised trials	serious[b]	not serious	not serious	not serious	none	473	443	-	SMD **0.12 SD lower** (0.24 lower to 0.01 higher)	⊕⊕⊕◯ Moderate[b]	CRITICAL
**Condomless sex events at 24-week follow-up**												
3	randomised trials	serious[b]	serious[d]	not serious	not serious	none	464	430	-	SMD **0.16 SD lower** (0.39 lower to 0.07 higher)	⊕⊕◯◯ Low[b,d]	IMPORTANT

CI: confidence interval; **OR:** odds ratio; **SMD:** standardised mean difference

Explanations

a. Analyze an article by dividing it into two sets of data and treating the article as two different studies
b. Some concern/high-risk bias.
c. A low number of study sample size.
d. High heterogeneity
e. Larger credible interval.
